# Reverse Osmosis Membrane Engineering: Multidirectional Analysis Using Bibliometric, Machine Learning, Data, and Text Mining Approaches

**DOI:** 10.3390/membranes14120259

**Published:** 2024-12-06

**Authors:** Ersin Aytaç, Noman Khalid Khanzada, Yazan Ibrahim, Mohamed Khayet, Nidal Hilal

**Affiliations:** 1 Department of Structure of Matter, Thermal Physics and Electronics, Faculty of Physics, University Complutense of Madrid, Avda. Complutense s/n, 28040 Madrid, Spain; eaytac@ucm.es; 2 Department of Environmental Engineering, Zonguldak Bülent Ecevit University, 67100 Zonguldak, Türkiye; 3 NYUAD Water Research Center, New York University Abu Dhabi, P.O. Box 129188, Abu Dhabi 129188, United Arab Emirates; noman.khanzada@nyu.edu (N.K.K.); ymi212@nyu.edu (Y.I.); 4 Chemical and Biomolecular Engineering Division, New York University, Brooklyn, NY 11201, USA; 5 Madrid Institute for Advanced Studies of Water (IMDEA Water Institute), Avda. Punto Com N° 2, 28805 Madrid, Spain

**Keywords:** reverse osmosis, Biblioshiny, Google Gemini, Flesch reading ease score, large language models, reading time score, technical term density, emotion analysis

## Abstract

Membrane engineering is a complex field involving the development of the most suitable membrane process for specific purposes and dealing with the design and operation of membrane technologies. This study analyzed 1424 articles on reverse osmosis (RO) membrane engineering from the Scopus database to provide guidance for future studies. The results show that since the first article was published in 1964, the domain has gained popularity, especially since 2009. Thin-film composite (TFC) polymeric material has been the primary focus of RO membrane experts, with 550 articles published on this topic. The use of nanomaterials and polymers in membrane engineering is also high, with 821 articles. Common problems such as fouling, biofouling, and scaling have been the center of work dedication, with 324 articles published on these issues. Wang J. is the leader in the number of published articles (73), while Gao C. is the leader in other metrics. *Journal of Membrane Science* is the most preferred source for the publication of RO membrane engineering and related technologies. Author social networks analysis shows that there are five core clusters, and the dominant cluster have 4 researchers. The analysis of sentiment, subjectivity, and emotion indicates that abstracts are positively perceived, objectively written, and emotionally neutral.

## 1. Introduction

Reverse osmosis (RO), a membrane-based separation process, has become a gold standard for desalination [1,2]. This technology uses hydraulic pressure as a driving force, and a semi-permeable membrane serves as a barrier for salt/water separation. The applied pressure forces the water molecules to pass through a dense polymeric semi-permeable or selective structure, while the dissolved impurities accumulate behind. In the current era, RO dominates > 60% of installed desalination capacity around the world [3]. In general, RO technology is one of the most important scientific fields handled by researchers in a very wide spectrum going from membrane engineering [4,5,6], modeling and optimization [7,8,9,10,11,12], membranes and modules recycling [13,14,15,16], renewable energy implementations [17,18,19], radioactive wastewater purification [20,21,22], treatment of RO brines [23], etc. The fabrication of RO membranes was first reported in 1959 by Reid and Breton using cellulose acetate (CA) polymer [24]. The CA RO membrane was synthesized to desalinate water. The membrane exhibited excellent NaCl rejection (98%) but showed significantly low permeance for water (<0.03 LMH/bar). Subsequently, Loeb and Sourirajan in 1963 developed a cellulose acetate membrane that successfully demonstrated improved water permeability and salt rejection (0.14 LMH/bar, 99%) [25].

Although the fabricated CA membranes performed well, their application remains restricted due to low thermal stability and chemical resistance, prompting researchers to continue searching for membranes with better thermal and chemical properties. Richter and Hoehn in 1971 created an aromatic hollow fiber polyamide (PA) membrane, which was the first non-cellulosic asymmetric membrane [26,27]. Despite having better chemical and biological stability and comparable rejection (99%), the water permeance of the PA membrane remained lower. In 1979, Cadotte made a significant breakthrough by introducing PA thin-film composite (TFC) membranes [28]. The inception of the TFC-RO membrane was one of the biggest technological advancements in the area of desalination and water treatment. The membrane revolutionized the desalination market, making RO economically feasible and applicable for large-scale applications [29]. The membrane was synthesized via interfacial polymerization (IP) using m-phenylenediamine (MPD) and trimesoyl chloride monomers. The IP process was carried out over a microporous polysulfone substrate, which was reinforced by a non-woven polyester fabric. The fabricated membrane exhibited greater water permeability (0.73 LMH/bar), comparable NaCl rejection (99%), and enhanced stability in both acidic and alkaline conditions when compared to the CA membranes.

However, when used for long-run desalination operations, the membranes encountered several shortcomings. Fouling formation on the surface [30,31,32,33,34], poor tolerance against free chlorine [35,36], and tradeoffs between water permeability and rejection are examples [37,38]. In addition, the TFC-RO membrane exhibits poor rejection against low molecular weight non-ionic hydrophilic contaminants, which are of emerging concern [39,40,41]. These stimuli researchers to develop new membranes or enhance existing membrane properties for sustainable desalination. Multiple endeavors have been undertaken over the years to improve the properties of RO membranes and facilitate sustainable operation. The adopted methods include the fabrication of RO membranes using co-solvent organic phase during IP [42,43,44,45], tailoring the support polysulfone structure [46,47,48], the addition of various functional nanomaterials in the top PA layer or polysulfone support layer [49,50,51], modification of membrane surface via polymers/nanomaterials deposition [52,53,54], and using novel monomers/polymers for RO membrane synthesis [55,56].

Bibliometric assessment is a quantitative analytical technique that uses mathematical, statistical, and sometimes machine learning (ML) methodologies to assess the association and impact of publications within a certain research subject. This type of study summarizes academic literature and highlights key documents, countries, authors, journals, and research institutions [57]. Based on bibliometric information, various study domains can be identified as having more scientific, exclusive, unique, and internal structure. This evaluation provides young scientists with views in their early stages of study and prepares them to contribute effectively to their area [58].

Artificial intelligence (AI) is a field of research that enables computers to have human-like intelligence. An important subset of AI is machine learning (ML), which intends to enable computers to learn from data through adaptive algorithms and carry out tasks such as decision-making and prediction [59,60,61]. Over the last decade, ML has made computer science and engineering more accessible to the public and contributed to new research [62,63]. Machine learning is classified into three main categories: supervised learning, unsupervised learning, and reinforcement learning [64]. In supervised learning, the computer collects patterns of labeled input and output data and uses them for predictions. In unsupervised learning, the computer analyzes unlabeled data and makes predictions [65]. In reinforcement learning, a computer learns to do a task through repeated tries and input from an external environment with a rewarding mechanism [66,67]. Natural language processing (NLP), another branch of AI, began in the 1940s with the creation of software models of language-recognized phrases, which enables computers to analyze, comprehend, and use human language and written text. NLP technology may be used for a variety of applications, including text summarization, sentiment/emotion/subjectivity analysis, information extraction, question answering, text clustering, machine translation, and many more [68,69,70,71,72].

Large language models (LLMs) are generative artificial intelligence systems that collect, process, translate, and respond to human language. They learn from large amounts of text using advanced computational methods (e.g., neural networks). They excel at language-based activities such as translation, sentiment analysis, and question answering. Unlike typical machine learning approaches, LLMs learn directly from raw text and do not require special features or specific domain expertise. This method enables LLMs to detect semantic relationships between various textual information. Commercially available LLMs (e.g., Claude 3.5 Sonnet, ChatGPT 4.0, and Gemini-1.5-pro) have demonstrated outstanding language generation capabilities [73,74,75,76].

While data mining is the process of discovering deep patterns in large chunks of data, text mining (TM) aims to extract and analyze important insights or patterns from irregular and unstructured text. TM is at the intersection of various fields such as data mining, machine learning, knowledge discovery, information retrieval, statistics, and natural language processing, and is essential in AI systems [77,78,79,80].

Summarizing this vast amount of research carried out in advancing RO membranes, identifying emerging trends in membrane surface engineering, and finding the roadmap for future advancement is a difficult task to accomplish in a single discourse. Examining published literature using bibliometric and data analytics approaches can be an efficient way to achieve this goal [81,82]. To direct resources and efforts toward study areas that have considerable potential to produce a deep influence, ultimately leading to advancements in the field, it is essential to carry out this kind of analysis [83]. It can offer a comprehensive assessment of the characteristics of research articles published, thus providing useful insights to researchers engaged in membrane manufacturing advancement. The efficacy of both bibliometric and data analysis techniques in extracting valuable insights from large volumes of RO membrane engineering research data is evidenced by the widespread application of these tools in other domains, which include progress in membrane water treatment technology [84], wastewater treatment [85], disinfection by-products in drinking water [86], capacitive deionization [87], adsorptive membrane [88], forward osmosis [89], etc. The approach enables researchers to examine the evolution of the field and identify key trends, approaches, and voids in the existing literature, thereby providing a roadmap for future studies, which is essential for the advancement of RO membrane manufacturing.

This study aims to encapsulate the six-decade evolution of RO membrane manufacturing and its advancement through published peer-reviewed studies with bibliometric (Bibliometrix-Biblioshiny tool (version 4.0), VOSviewer (version 1.6.20)) and machine learning (Python, R) software (Python 3.13.1). By examining both historical and contemporary research, this study aims to provide a comprehensive overview of advancements in RO membrane manufacturing and its surface engineering, assess their impact on membrane performance, and provide information to help researchers direct their own work and develop more effective research strategies. The information supplied by this article can also be useful for new researchers entering the field in terms of allocating research funds, developing training programs, and identifying new areas of research.

## 2. Data, Software, and Methods

The data for this study were acquired through a search conducted on the Scopus database as of 11 March 2024. Criteria for selecting the Scopus website for analysis include its larger size, its coverage of more titles indexed by other large databases, and its comprehensive source index that can export metadata and published data from a wide range of study fields. Additionally, most of the scientific output is published in English in this database, and it is acknowledged as a trustworthy repository [90,91]. The keywords, which are used to search in the title, abstract, and keywords fields of the articles, have a wide range to cover all the necessary literature. The rationale behind using so many keywords is to include different aspects of RO membrane engineering from core concepts to emerging notions. The search keywords target the fundamental processes, design, development, and optimization, commonly used materials, specific types of membrane configurations, ways of altering the surface properties, capturing innovative research approaches, specific types of membranes, key techniques, common monomers, the usage of nanotechnology, membrane customizing-related words, etc. The keywords used for the search can be seen in Table 1.

To ensure that the search results are specifically related to reverse osmosis technology, the keyword “reverse osmosis” was incorporated into the search query using the AND operator. Further refinement criteria were applied during the search stage to improve the consistency of the analysis and the quality of the results, including “journal” as the source type, “article” as the document type, “final” as the publication stage, “English” as the language, and excluding publications from the year 2024. Following the extraction of data from the Scopus website, a manual screening process was conducted, resulting in a final dataset comprising 1424 entries. A combination of VOSviewer (version 1.6.20), Biblioshiny (version 4.2.3), R, and Python was utilized for the analysis. For better presentation, the results were then illustrated in different visualization tools.

Guido van Rossum began implementing the Python language in late 1989 at the Netherlands’ Centrum Wiskunde & Informatica (National Research Institute for Mathematics and Computer Science). Today, Python has become a popular programming language for statistical and machine learning due to its extensive community, frameworks, and libraries [70,92]. R is a programming language for statistical computing and data visualization [93]. Bibliometrix(version 4.0) (Biblioshiny tool in the R interface) is an open-source application to undertake thorough scientific mapping analysis, which supports a suggested procedure for conducting bibliometric studies [94]. VOSviewer is publicly downloadable software for creating and viewing bibliometric maps, focusing on their graphical depiction [95]. Various calculations were performed to examine the dataset, and all equations and parameters are listed in Supplementary Note 1. These include global citations (GC), total citations (TC), local citations (LC), compound annual growth rate $\left({CAGR}_{C}\right)$, co-authors per document (${cAD}_{C}$), document average age in a collection $\left({DAA}_{C}\right)$ or an item $\left({DAA}_{It}\right)$, average GC per document of the dataset ${(AGCD}_{C})$ or an item $({AGCD}_{It}$), average GC per document published in the corresponding year of the collection $\left(AGCD_{C_{y}}\right)$, average normalized global citations per document published in the corresponding year value of the collection ${(ANGCD}_{C_{y}})$, global citations per year of a document $\left({GCY}_{i}\right)$, relative global citations of a document published in a particular year $\left({RC}_{i_{y}}\right)$, average relative global citations value of an item $\left({ARGC}_{It}\right)$, international co-authorship ratio of the dataset (${IcA}_{C}$), articles fractionalized value of an author (${AF}_{au}$), *h*-index, *m*-quotient (or *m*-index), *g*-index, Flesch reading ease score (FRES) including interpretations of the scores based on Table S1, technical term density percentage (TTD %), exact match (EM), cosine similarity (CS), and cosine distance (CD) [80,81,82,96,97,98,99,100,101,102,103,104,105,106,107,108,109,110,111,112,113,114,115,116,117,118,119,120,121,122,123,124,125,126,127,128]. 

## 3. Results and Discussions

### 3.1. RO Membrane Engineering Statistics

It is of great importance to present basic statistics before proceeding to the elaboration of the RO membrane engineering dataset. Basic statistics provide the reader with a preliminary knowledge of the subject by providing a general framework of the event. This helps to understand the scope and context of the research. Essential statistical data provides a better understanding of the research methodology and findings. Table S2 indicates the essential information about the RO membrane engineering collection.

**Figure 1:**
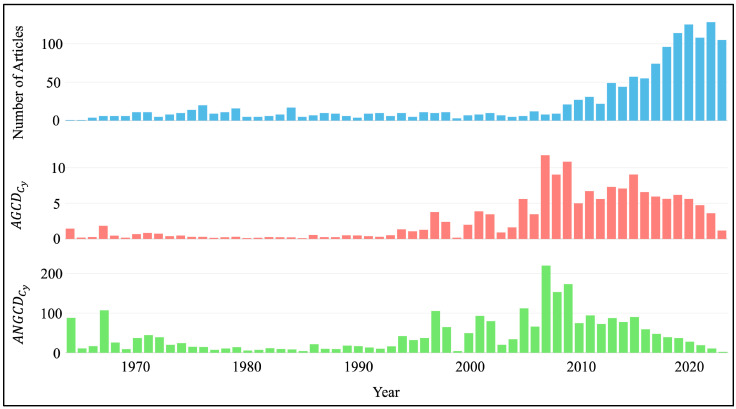
Yearly publications, average global citations per document published in the corresponding year $\left({AGCD}_{C_{y}}\right)$ and average normalized global citations per document published in the corresponding year $\left({ANGCD}_{C_{y}}\right)$ results of the collection.

The reverse osmosis membrane engineering dataset covers articles published between 1964 and 2023 in 225 different journals. The $\left({CAGR}_{C}\right)$ in this field is 8.21%, and the average age of the documents $\left({DAA}_{C}\right)$ is 14 years. Each document has received an average of 41.75 citations ${(AGCD}_{C})$. Authors have benefited from 38,424 references. The authors’ keywords consist of 1987 different terms. There are a total of 3110 authors in the dataset, with 42 single-author documents written by 39 different authors. Each document has an average of 4.79 co-authors ${(cAD}_{C}),$ and the international co-authorship rate ${(IcA}_{C})$ is 21.07%. These statistics show that research in the field of reverse osmosis membrane engineering is widely distributed, and international collaborations play an important role. In addition, high citation rates reveal that studies in this field are of great interest and valued by the scientific community. These data emphasize the importance of reverse osmosis technology in water treatment and desalination processes, as well as the continuous development of research in this field. Time series of annual publications, average global citations in the corresponding year and average normalized global citations in the corresponding year were also created to better understand the continuity and trend of studies on RO membrane engineering (Figure 1). These time series allow for a more detailed analysis of developments and changes in the research area. Please note that the numbers in Figure 1 are on a yearly basis, not cumulative.

When the annual publications graph in Figure 1 is examined, the topic of RO membrane engineering began with the first paper published in 1964 titled “Cellulose acetate membranes: Electron microscopy of structure”, published by Riley et al. [129]. The popularity of the topic really took off in 2009 with 21 publications, and the number of scientific researches has accelerated with increasing momentum. The number of articles published in 2023, the last year of the dataset, is 105. When average global citations per document published in the corresponding year ${(AGCD}_{C_{y}})$ and average normalized global citations per document published in the corresponding year ${(ANGCD}_{C_{y}})$ values are considered in Figure 1, it is seen that 8 articles published in 2007 are the studies with the highest impact in the field. The average ${AGCD}_{C_{y}}$ and ${ANGCD}_{C_{y}}$ values of the articles published in this year reached 248.50 and 3.81, respectively. The reason all these metrics are high is that 2007 was a breakthrough year for RO membranes, and the publications made in this year had a high impact on the field. In 2007, Byeong-Heon Jeong, Eric M.V. Hoek, Yushan Yan, Arun Subramani, Xiaofei Huang, Gil Hurwitz, Asim K. Ghosh, and Anna Jawor introduced an innovative production method for reverse osmosis membranes, which would later be called the thin film nanocomposite (TFN) membrane [128]. This novel approach significantly increased membrane flow while preserving comparable solute rejection to the previously manufactured thin-film composite (TFC) membrane. This boost in permeability is attributed to the super-hydrophilic molecular sieve nanoparticle holes, which create separate routes for flow [130]. The production of this innovative membrane has shifted the interest of membrane engineers to TFN membranes, and the number of researches on this subject has increased (more details of publications on TFN membranes will be mentioned in the following sections). The article published by Jeong et al. (2007) has been widely cited, which has increased the ${AGCD}_{C_{y}}$ and ${ANGCD}_{C_{y}}$ values of publications in 2007. Besides, the impact of the other seven papers published in 2007 on these metrics should not be forgotten. These seven papers have an average citation count of ~129 and include significant papers investigating the effects of commercial RO membranes with different surface properties on fouling by bovine serum albumin (BSA) and sodium alginate [131], and developing an innovative method for surface modification of TFC membranes, such as grafting with poly(ethylene glycol) [132]. The distribution of the number of pages, number of references, and number of citations of the articles in the dataset is shown in Figure S1, accompanied by relevant discussions in Supplementary Information Note 3.

**Figure 2:**
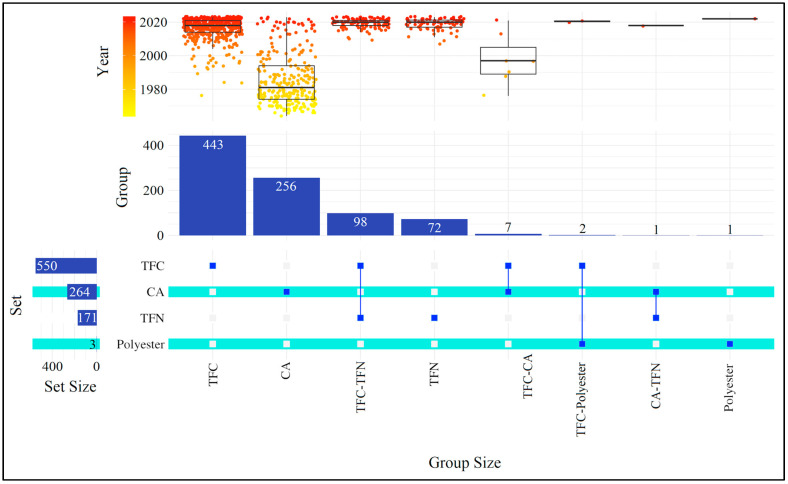
Classification of publications in terms of used polymeric material.

Polymeric semipermeable membranes have had controlled commercial uses since the earliest days of RO desalination plants. Due to their technical maturity, polymeric membranes are easy to work with, economical to produce, and offer increased permeability and salt rejection efficiency [29]. Actually, polymeric RO membranes do not entirely reject pollutants, and water permeability may always be increased. Although large organic compounds (i.e., isoxathion, pesticide) and ionic contaminants (i.e., sodium chloride) have been successfully rejected, it is more difficult to achieve high rejection for small neutral organic compounds (i.e., methanol, ethanol, 2-propanol, and urea) [133]. The structural and chemical features of these membranes control water flow, salt rejection, fouling resistance, and chemical stability, all of which have a significant influence on energy consumption and costs in the process [134]. Innovations to reverse osmosis technology are usually targeted at increasing water flux and salt rejection while reducing fouling and energy consumption. Many breakthroughs have been achieved in RO, including water pretreatment, module design, and energy recovery [133]. The milestones of polymeric membranes in reverse osmosis are as follows: Reid and Breton developed hand-cast symmetrical cellulose acetate (CA) membranes in the late 1950s, but their permeate flux was low despite retaining 98% of salts on the membrane surface. Loeb and Sourirajan developed an asymmetric membrane that improved water flux. This led to increased interest in membranes for desalination and substantial studies into high water flux and salt rejection. Since 1969, CA was regarded as the best form of polymer. However, Richter and Hoehn invented non-cellulosic asymmetric membranes (aromatic polyamide hollow fiber membrane), which were eventually commercialized by DuPont. Although this membrane had poor water flow and salt rejection, it was more stable, durable, and versatile compared to CA membranes. Francis pioneered the thin-film composite (TFC) membrane by casting thin-film CA over a water surface, then annealing and laminating it to a CA support. Later, Cadotte and Peterson discovered efficient TFC membranes, and ongoing research continues to improve their performance in areas like chlorine resistance and antifouling [135]. However, the most significant advances have come from upgrading membrane materials [133]. Since there is a variety of polymeric materials used in RO processes, in the next step of our study, a classification of publications in terms of used polymeric material was conducted. This type of analysis allows us to find out the orientation of RO researchers and the popularity of polymeric material. The resulting upset graph can be seen in Figure 2.

As can be seen in Figure 2, thin-film composite (TFC) membrane is the first choice of membrane engineers in reverse osmosis. A total of 550 articles mention TFC membranes, and 443 of these articles solely include this type of polymeric material. Since Cadotte and colleagues [28] proposed the technique of interfacial polymerization (IP) to create polyamide (PA) thin-film composite membranes, subsequently developed products have mainly dominated the desalination membrane market, with spiral wound configurations accounting for more than 90% of market sales [134]. These membranes are generally composed of three basic layers: a bottom nonwoven fabric layer (~100–150 μm), a middle finely microporous support layer (~50 μm), and a top ultrathin barrier layer (~0.01–0.2 μm) [135]. The porous support enables the rigidity that is essential for the entire membrane structure to work under high pressures, while the ultrathin top layer is the primary water filtering component [136]. The IP process to form the top ultrathin barrier layer occurs with the following reaction: the two types of reactants, nucleophilic (i.e., amines and alcohols) and electrophilic (i.e., acyl chloride), dissolved in incompatible phases (mostly aqueous and organic solution) [137]. Aside from IP, coating methods like photo-grafting, dip-coating, electron beam irradiation, and plasma-initiated polymerization are also used to attach an ultrathin barrier layer to a support membrane [138]. In TFC membranes, the selective layer and support might be separately adjusted to reach the required performance. Many kinds of polymers have been introduced as top ultrathin layers, such as polyamide (PA), polyvinyl methyl ether (PVME), styrene-acrylonitrile copolymer (SAN), and polyurethanes (PU). Due to the ease of fabrication, stability, and thermal resistance, polyethersulfone (PES), polysulfone (PSU), poly(phthalazinone ether sulfone ketone) (PPESK), polycarbonate (PO), polyetherimide (PEI), polyacrylonitrile (PAN), polypropylene (PP), and polyphenylene oxides (PPO) are commonly used as support layers [135]. The most common combinations for the top layer and support layer are PA-PES or PA-PSU [139,140]. One drawback of the TFC membrane is compaction. In TFC membranes, while pressure provides a driving force or creates a chemical potential for transport, it also causes the microporous support polymer to lose porosity over time, resulting in decreased flow, which is called compaction. Compaction causes an intrinsic flux drop, which requires greater operating pressure to maintain target flux levels, resulting in increased energy consumption [141]. Over the last decades, TFC membranes have been continuously enhanced to improve their performance regarding permeate flow, salt, and pollutant rejection, as well as increased resistance to membrane fouling and chlorine [142]. The most important factors affecting TFC membrane formation are pore size, monomer concentration, nanomaterials (if used), additives and surfactants, choice of organic solvent, substrate porosity, reaction temperature and time in interfacial polymerization, and hydrophilicity [143]. Membrane engineers dealing with the fabrication of TFC membranes have conducted their work in a wide range of fields. IP is the main technique for TFC membrane fabrication, and some researchers aim to enhance this technique with different applications such as IP assisted with an aromatic/aliphatic organic solvent mixture [144], sequential process of blade coating-spraying-IP [145], addition of dimethyl sulfoxide in the IP media [146], ionic liquid-mediated IP [147], tannic acid reinforced IP [148], cosolvent-assisted IP [149], IP with UV-introduced photo-fries rearrangement [150], layered IP [151], in situ free IP [152], and more. Membrane engineering specific to the pollutant to be removed constitutes another topic in TFC membranes. Trace organic contaminants elimination [153], desalination [154], chromium(VI) removal [155], boron remediation [156], arsenic(III) removal [157], pesticide treatment [158], etc. are a few examples of pollutants that RO membrane engineers deal with. Not only the production but also the characterization of TFC membranes is an important task in the field of RO membrane engineering. Fourier transform infrared spectrometer (FT-IR) [159], scanning electron microscope (SEM) [160], atomic force microscope (AFM) [161], thermogravimetry [162], contact angle measurement [163], etc. can be used for the characterization of developed TFC membranes.

Figure 2 indicates that the second most researched polymeric material is cellulose acetate (CA) (mentioned 256 times alone, 264 times in total). This polymer is built via incorporating the acetyl radical of acetic acid into cellulose (wood or cotton) [164]. CA is commonly preferred by RO membrane engineers because of its accepted properties during the fabrication process, such as good mechanical strength, high hydrophilicity, low protein adsorption, perfect transport characteristics, superb film-forming properties, low cost, proper solubility in some common solvents, and biodegradability. However, there are also some negative sides of CA membranes. The presence of nonreactive functional groups leads to poor thermal, chemical, and mechanical resistance, making them unsuitable for affinity-based adsorption separation. CA membrane’s thick skin layer and low sublayer porosity result in minimal flux during applications [165,166,167]. Furthermore, CA membranes lack robustness and are readily contaminated in nature [168]. However, recent studies indicate that CA membranes are less prone to fouling compared to typical PA membranes [165]. The phase inversion (PI) approach is the most popular method for CA membrane synthesis, and the membranes produced often have finger-like, sponge-like, or both porous features [169]. This process involves dissolving a polymer and a porogen in a dope solution (solvent). The solvent is then cast on a glass plate, and the resulting film is placed in a coagulation bath. Solvent and nonsolvent exchange happen, resulting in the phase inversion process. With this technique, a flat sheet polymeric membrane is formed. The same approach is used to create hollow fibers by extruding the dope solution [164]. Other techniques to prepare CA membranes are immersion precipitation and thermally induced phase separation (TIPS) [169]. CA membranes can be modified with different materials (carbon nanotubes (CNTs), graphene oxide (GO), and metal oxides) to improve water permeability and surface porosity and to give them antibacterial and photocatalytic characteristics [166]. The CA membrane engineering topic offers a diverse variety of applications. Membranologists have used innovative approaches to improve the performance of cellulose acetate membranes, such as the grafted/crosslinked method [170], electron spin approach [171], and surface-initiated polymerization technique [172]. There are also theoretical studies in the RO CA membrane engineering domain, including the use of the Taguchi method [173], combined nonlinear membrane transport and film theory model [174], and finely porous model [175].

Thin film nanocomposite (TFN) membranes are the third most mentioned type of polymeric material in the collection. TFN membranes are mentioned in 171 articles in total, with 72 of these being articles containing only TFN membranes. Thin film nanocomposite membranes were first introduced by Jeong et al. (2007) [128] in a paper titled “Interfacial polymerization of thin film nanocomposites: A new concept for reverse osmosis membranes” in the *Journal of Membrane Science*. Although this research group prepared a similar membrane in 2005 and presented it at the AIChE Annual Meeting and Fall Showcase in the United States, this membrane was referred to as Tfnc rather than TFN [176]. TFN membranes are TFC membranes incorporated with nanomaterials. These nanomaterials could be MXene, graphene, graphene oxide (GO), covalent organic frameworks (COFs), metal–organic frameworks (MOFs), boron nitride, etc., and they create nanochannels to increase water permeation, enhance anti-fouling ability, improve chlorine resistance, increase mechanical, thermal, and chemical stability, and provide antibacterial performance [177,178,179]. Integration of nanomaterials into TFC membranes to create TFN membranes can be done in three ways. The first is dispersing the nanomaterials into the water/organic phase of monomers, which results in them being randomly captured and bound by the PA structure through the IP process (TFNa). The second method is dispersing the nanomaterial into the substrate structure while using the phase inversion method (TFNs), and the last procedure involves uniformly depositing the nanomaterial onto the porous substrate before the IP process (TFNi) [178]. In the dataset, the RO TFN membrane engineering studies include an extensive spectrum. In addition to common nanomaterials such as carbon nanotubes [180], graphene oxide [181], covalent organic frameworks [182], and metal–organic frameworks [183], niche and cutting-edge nanomaterials are also integrated into the production stage. This include polyvinyl alcohol/titanium silicate-1 [184], the mobile composition of matter-41 (MCM-41), and santa barbara amorphous-15 (SBA-15) (two kinds of mesoporous silica) [185], hydrophobic methyl trichlorosilane (MeSiCl_3_) [186], cyclodextrin [187], and many more. One of the most preferred nanomaterials by researchers is carbon derivatives. Among the studies with carbon derivatives are graphitic carbon nitride/polypyrrole [188], carbon nanotubes [189], 1D/2D graphitic carbon nitride (g-C_3_N_4_) nanohybrids [190], carbide-derived carbon [191], and carbon nitride [192]. It is obvious that silica/silica composites [193,194,195,196] and zeolite/zeolite composites [197,198,199,200] are preferred to create TFN membranes.

Polyester membranes are a new topic for RO membrane engineers, and as shown in Figure 2, the number of articles mentioning them is limited (three papers in total, with one paper alone mentioning polyester). Polyesters are regarded as one of the oldest types of polymers analyzed synthetically, and they are one of the most significant usable polymers in industrial applications today [201]. Polyesters are a class of polymers with repeated ester groups as the backbone of the primary chain structure [202]. Synthetic polyesters are produced by reacting a dicarboxylic acid with a diol or by self-condensation of an ω-hydroxy acid [203]. The use of polyester in membrane matrices is mostly implemented either by integration with a barrier layer or an additional new layer on top of the barrier layer, which mostly means modifying a TFC membrane [54,56,204].

Another important result in Figure 2 is the high number of papers in which TFC–TFN membranes are discussed together (98 articles). Most of the time, researchers produce or buy commercially available TFC membranes, then add various nanomaterials to these membranes, converting them into TFN membranes, and examine and compare the performance of the two membranes produced [205,206,207,208,209]. Creating CA-based TFC or TFN membranes or comparing CA membranes with TFC or TFN membranes has been conducted by researchers in a few papers (seven and one article respectively) [210,211,212].

The box plots at the top of Figure 2 represent the column-based (group-based) publication years of the articles. When publishing year distributions are examined (only the top four groups with high data density), it is clear that RO membrane engineers have been researching TFC–TFN membranes to compare the performance of both polymeric materials in recent years. The average publishing year of the articles in which the names of TFC–TFN membranes appeared together was 2019.3 (median value = 2020). It is also understood that TFN membranes have become popular in recent years. The average publishing year of articles containing only TFN membranes is 2018.9 (median value = 2020). TFC membranes have started to fall out of favor with a value of 2016.5 (median value = 2018). The average publication year of the articles on CA membrane is the lowest, with 1985.7 (median value = 1981). This situation shows that CA membranes are almost outdated in RO membrane processes, and research has come to an end. Since the number of articles in other groups is low, we believe that statistical evaluation is not correct.

**Figure 3:**
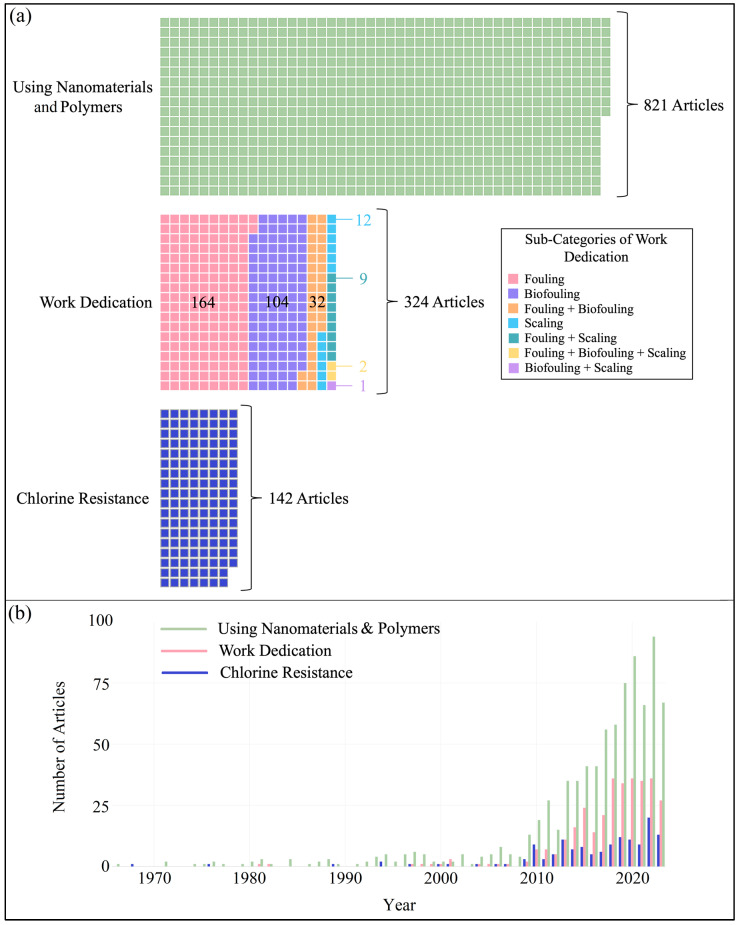
(**a**) Surface-engineered RO membranes and their work devotion and (**b**) publication years of corresponding articles.

To combat the challenges encountered by RO membranes, researchers have conducted substantial research on various materials and ways to improve membrane characteristics. The use of novel nanomaterials (metal oxides, zeolites, carbon-based nanoparticles) and polymers/zwitterionic polymers (polyvinylchloride (PVC), polyvinyl alcohol (PVA), polyampholytes, polybetaines, poly(ethylene glycol), polyethyleneimine) has demonstrated enhancements in several properties of RO membranes, such as water permeability, chlorine resistance, antifouling capabilities, and antibacterial features. Their incorporation has also been shown to enhance the mechanical strength and thermal stability of RO membranes. The performance gain can be ascribed to mechanisms including increased surface area, improved interfacial interactions, modification of surface characteristics, and enhanced structural integrity conferred by the inserted nanomaterials or polymers. The properties of these engineered membranes are significantly influenced by the type, concentration, chemical characteristics, and dimensions of the embedded/functionalized nanoparticles/polymers [213,214,215]. Thus, the properties of nanocomposite membranes can be tailored based on the specific nanomaterial utilized. These polymers/materials can be incorporated into thin-film composite (TFC) membranes via several fundamental methods. The diverse integration procedures significantly influence the development of TFC membranes and are crucial factors to consider when evaluating their effectiveness. The increasing interest in the development of nanomaterial/polymer-modified RO membranes is evident from the rising number of published research works. Figure 3a represents the quantitative data for engineered RO membranes involving different polymers and nanomaterials (821 articles). Graphene oxide (GO) is one of the most popular materials among RO membrane researchers. GO consists of a single layer of graphite oxide and is typically created by oxidizing graphite, then dispersing and exfoliating it in water or other appropriate organic solvents [216]. GO-integrated RO membranes have found their place in the literature significantly [217,218,219,220]. Carbon nanotubes (CNTs) are cylindrical nanostructures made up of carbon atoms organized in a hexagonal lattice, with exceptional mechanical, electrical, and thermal characteristics [221]. Depending on the existence of the layer, CNTs are classified into two types: single-walled carbon nanotubes (SWCNTs) and multi-walled carbon nanotubes (MWCNTs) [222]. The use of CNTs to enhance the performance of the RO process is promising for improving the overall efficiency of water treatment, and membranologists are focusing on this area as well [223,224,225]. Zeolites are porous hydrated aluminosilicates with a three-dimensional structure that includes cations of alkaline elements, alkaline earth metals, and other monovalent or multivalent metals. Because of their distinct structure, which comprises large open spaces and channels, zeolites display features characteristic of nanoporous materials and have the potential to shed and absorb water in amounts greater than 30% of their dry weight [226]. Due to these superior properties, zeolites have also taken their place in research to contribute to the RO process [227,228,229]. Silica nanoparticles (SiNPs) are made up of silicon dioxide, the most prevalent substance on Earth. They are particularly appealing because of their simplicity of synthesizing, colloidal rigidity, tunable particle size, biocompatibility, ease of surface functionalization, and potentially scalable manufacture [230,231]. The implementation of silica nanoparticles into the membrane matrix is another type of study that provides new insights for researchers [232,233,234]. Layered double hydroxides (LDH), additionally referred to as hydrotalcite-like systems or anionic clays, have received a lot of interest since they qualitatively resemble ordinary intercalation compounds. One of the benefits of LDH is the wide range of potential compositions and metal-anion combinations that can be manufactured. Aside from that, it possesses unique properties such as strong biocompatibility, high chemical stability, pH-dependent solubility, etc., making it a sought-after material. Therefore, membranologists have also taken up the study of LDH in membrane engineering [235,236,237]. The exceptional physical, chemical, and biological features of silver nanoparticles (AgNPs) have been the primary subject of investigation in RO membranes. Silver compounds and silver ions are generally known as potent antibacterial agents [238]. For this reason, AgNPs are widely employed in RO membrane fabrication for antibiofouling, antimicrobial activity, and water disinfection [239,240,241]. Polyvinyl alcohol (PVA) is an odorless, biocompatible, and nontoxic synthetic polymer with O_2_ and scent barrier properties [242,243]. By grafting PVA onto the RO membrane surface, the researchers expected to reduce the chlorine-sensitive regions of amide linkages and terminal amino groups in aromatic polyamide chains. The grafted PVA layer, which is also bonded to the membrane surface, can be expected to form a hydrophilic protective layer above the active layer, preventing the accumulation of contaminants and chlorine attack on the active layer [244,245,246]. Polydopamine (PDA) is a dopamine-derived artificial eumelanin polymer with catechol, imine, and amine functional groups. PDA has qualities like mussels and can strongly connect to varied substrates with high binding strength, including wet surfaces [247]. The usage of PDA in membrane engineering can be found in the following articles [248,249,250]. Apart from these predominantly used materials, there are also niche materials preferred by membrane engineers, including polyvinylchloride (PVC) [213], nanodiamond [251], Ti_3_C_2_T_x_MXene [252], metal–organic frameworks [253,254], and carbon dots [255] to enhance membrane performance. Instead of using only nanomaterials or only polymers, RO scientists have also worked on improving the performance of the reverse osmosis process by utilizing the different properties of both types of materials. These studies include polyethyleneimine-GO [256], TiO_2_-GO [257], CNT-GO [258], cellulose fiber-CNT [259], GO-zeolite [260], GO-AgNPs [261], GO-PVA [262], cerium(IV)-PVA [263], PDA-curcumin [264], or PDA-nano copper [265].

Fouling in membrane processes happens when dissolved and particulate debris in feed water settles on the membrane surface, increasing the total membrane resistance [266]. Reverse osmosis membrane fouling is a major impediment to consistent membrane function. Membrane fouling may considerably impair productivity and permeate quality while raising operation costs owing to higher energy consumption, extra pretreatment, foulant removal, and membrane cleaning and maintenance, as well as a decrease in membrane lifetime [267]. Biofouling (a special kind of fouling) is commonly described as “the Achilles heel” of reverse osmosis membrane separation. Biofouling, commonly referred to as biological fouling, is the deposition, development, and execution of metabolic processes by microorganisms on the membrane surface that impedes the attainment of technical, aesthetically attractive, or economically desired objectives [268,269]. The yearly cost of preventative actions to reduce biofouling phenomena in the desalination sector is around USD 15 billion globally [270]. Scaling is another problem that membrane engineers have to face. Scaling is another type of fouling phenomenon that is caused by the deposition of ions on the membrane surface [271]. Mg^2+^, Ca^2+^, Al^3+^, Ba^2+^, SO_4_^2−^, and CO_3_^2−^ are the common scaling ions [272]. Figure 3b shows the yearly distribution of published papers on engineered RO membranes prepared with different polymers and nanomaterials. The figure indicates that this topic did not receive much attention from 1966 to the mid-1990s. After 1993, researchers began focusing on this topic, and RO membranes engineered with different polymers and nanomaterials became one of the main research areas, with 13 papers published in 2009. Since 2017, more than 50 studies have been published each year, with 2022 recorded as the year with the most articles published, with 94 articles.

As shown in Figure 3a, work dedication, a large portion of the conducted studies focused on resolving the fouling issue associated with RO membranes. Of 324 research articles describing the work dedication, 164 studies were dedicated to fouling. These studies have a wide variety of applications, such as creating a membrane by controlled-release sulfate radical modification [273], preparing sulfonated membranes [274], fabricating diazotized membranes on commercial polyethylene textile [275], tailoring polyethyleneimine-based membranes [276], and applying a new modification technique for fabricating TFC membranes [277], etc. In addition, special attention has been paid by the research community to investigating biofouling behavior (only biofouling related papers are 104). This can be attributed to the dominance and complex nature of biofouling in the RO process. Complexation of tannic acid-AgNPs on PA TFC membranes [278], modification of TFC membranes by chitosan-Ag particles [279], construction of pseudo-zwitterionic PA membranes [280], biocidal surfactant-assisted fabrication of TFC membranes [281], modifying membranes by a facile method [282], etc. are in the scope of researchers to gain the upper hand against biofouling. In contrast, fewer articles were identified using the keywords “scaling”, “fouling + scaling”, biofouling + scaling”, and “fouling + biofouling + scaling”. Figure 3b refers to the annual changes in the number of papers published about work dedication. After the first article was published in 1981, it is obvious that there was not much interest in this topic until 2010. Since that year, membrane researchers have accelerated membrane fouling/biofouling/scaling studies, with more than 30 studies appearing in the scientific literature each year between 2018 and 2022.

At this point in the study, we have revealed the number of articles on chlorine resistance, which is a very important sub-classification. Producing a TFC/TFN PA membrane with chlorine resistance is very important for simplifying pretreatment operations and lowering operational costs. In RO systems, the injection of chlorinated chemicals (i.e., Cl_2_, ClO_2_, NH_2_Cl, NaClO) into the feed solution is regarded as a critical step to avoid membrane fouling caused by microorganisms. Because residual active chlorine is highly oxidizable, the secondary amide structure of the PA framework is vulnerable to active chlorine engagement via reversible amide N-chloride substitution, followed by irreversible Orton rearrangement, resulting in harm to the PA membrane’s framework and deterioration of membrane performance. To avoid impact on the membrane structure, the de-chlorination procedure must remove most of the active chlorine. However, this extra operation greatly increases operating costs, and it is still impossible to avoid structural degradation caused by active chlorine [283,284,285]. As seen in Figure 3a, 142 studies focused directly on increasing chlorine resistance or indirectly achieved an increase in chlorine resistance. These studies include extensive applications. He et al. (2023) [286] prepared a chlorine-resistant PA membrane using organic-organic interfacial polymerization, Li et al. (2022) [287] created the TFC RO PA membrane with tri-acyl chloride containing thioether units, and Shalaby et al. (2022) [288] used physical irradiation surface treatment for this purpose. Vatanpour et al. (2022) [289] utilized infinite coordination polymer (ICP) modification of the TFN PA membrane to enhance chlorine resistance while increasing antifouling. Idrees and Tariq (2022) [290] employed different surface and structure modification strategies for enhancing chlorine resistance in PA membranes. Sharabati et al. (2022) [291] created a zwitterionic polysiloxane-PA hybrid active layer to create a chlorine-resistant TFC membrane while ensuring high performance. Figure 3b shows the yearly distribution of studies on chlorine-resistant membrane production. The first study on chlorine-resistant membranes was recorded in 1968, and for a long time (until 2010), the engineering of this type of membrane remained at a low level. In 2010, with the publication of 9 articles, researchers realized the importance of this subject, and momentum for this type of research increased in the following years. The year 2022 is identified as the year with the highest number of scientific activities on the production of chlorine-resistant membranes, with 20 articles.

### 3.2. Important Authors

**Figure 4:**
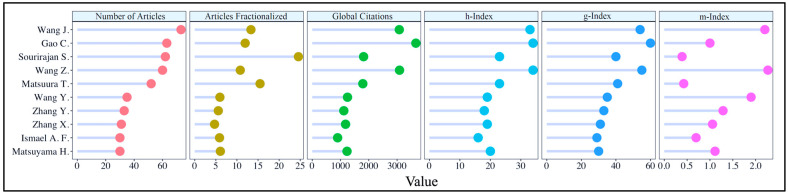
Important metrics of top 10 scientists in the reverse osmosis membrane engineering domain based on the number of publications.

Identifying prominent authors in a field may help readers stay up-to-date on new discoveries, find reliable sources of inspiration, and discover new perspectives and ideas [97]. Authors working in the field of reverse osmosis membrane engineering play a critical role in the development of water treatment and desalination technologies. The authors design innovative technologies that treat seawater and other polluted water sources into drinking water, help reduce environmental pollution, prioritize the protection of ecosystems, create sustainable environmental management, increase energy efficiency, reduce operating costs of RO technologies, and improve the welfare of societies by ensuring the safe and sustainable use of water resources through their research and development efforts. For all these reasons, it is important to highlight scientists working on RO membrane engineering studies. Figure 4 reveals the important indicators of the top 10 authors in the field based on the number of articles they published and their corresponding metrics.

The bibliometric results in Figure 4 indicate that Wang J. has the highest number of publications in the RO membrane engineering domain (73). While Gao C. published fewer articles (63) than Wang J., he is one of the most influential scientists in this field, with an *h*-index of 34, a *g*-index of 60, and a global citation count of 3664. Wang Z. ranks at the top of this list considering the *m*-index value (2.267). It is also seen that the *h*-index value of Wang Z. is very high (34). At this point, it is necessary to dedicate a special paragraph to a name in the top 10, Prof. Sourirajan Srinivasa. Prof. Srinivasa Sourirajan is well-known in the desalination and membrane communities as the father of the reverse osmosis method. This began in 1960 at the University of California, Los Angeles when he and Sidney Loeb presented the first cellulose acetate membrane for saltwater desalination. Following the invention of this semipermeable membrane, membrane science and technology research has expanded tremendously, with the discovery and use of various new advanced materials in membrane engineering. Prof. Sourirajan’s breakthrough findings have had a significant impact on today’s water, food, and sanitation systems. Only being the leader in the articles fractionalized metric (24.58) is not enough to express Prof. Sourirajan’s place in membrane science. He served as a role model for individuals and future generations interested in reverse osmosis, membrane engineering, desalination, and water treatment. His studies in desalination and water treatment influenced subsequent membrane scientists and researchers [292]. Prof. Takeshi Matsuura, another name in the top 10 authors list, is also a scientist in the field of reverse osmosis whose name should be mentioned. Prof. Matsuura was both a student and one of the closest colleagues of Prof. Srinivasan; together they conducted collaborative studies on reverse osmosis membranes. His research has focused on membrane transport during reverse osmosis operations and the creation of cellulose acetate membranes for reverse osmosis technologies. His other achievements include innovative water treatment applications using nanofiber membranes, the use of macromolecules to accomplish surface modification of polymer membranes, and the surface characterization of membranes using atomic force microscopy-based approaches. Prof. Matsuura’s research has been extremely practical, yielding valuable insights into the design and characterization of membrane systems. As a result, he has inspired numerous scientists, technologists, and engineers working in the desalination and water processing industries [293].

**Figure 5:**
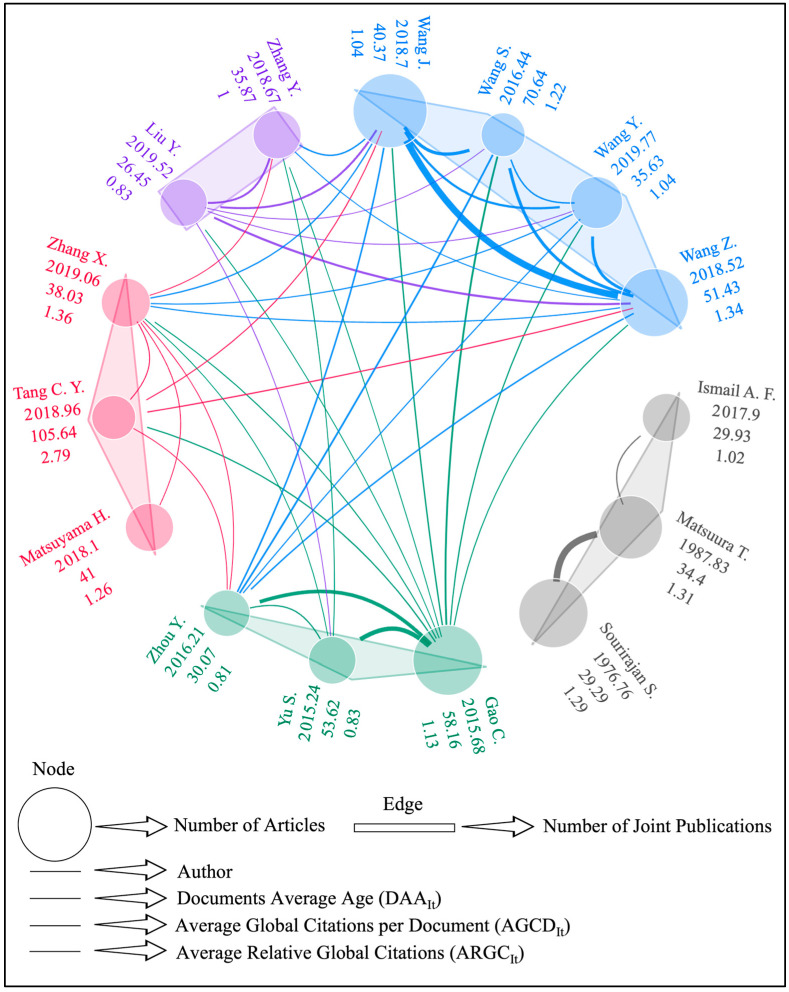
Co-authorship analysis of the authors (weights = documents, min. number of documents of an author = 25, clusters with single items removed).

Collaboration and social networking are extremely important in the scientific community for a variety of reasons. Scientists can interact more rapidly and efficiently through social networks. Scientific collaborations also allow for more complete and in-depth study by exchanging information and resources from many areas of expertise, increasing the repeatability of scientific investigations, and improving the accuracy and dependability of results. Additionally, young scientists can engage with established researchers, receive mentoring, and further their careers via social networks. In Figure 5, the collaboration network of the authors (co-authorship analysis) can be viewed. Please note that for the clarity and interpretability of the result, an author’s minimum number of papers is restricted to 25.

In Figure 5, each color denotes a cluster, and the size of the node (circle) is directly proportional to the number of articles of the author. Likewise, the thickness of the edge (line) denotes the number of joint publications of the authors. As Figure 5 depicts, there are five core research groups working on the RO membrane engineering domain, with two to four researchers. While the gray cluster with Sourirajan S., Matsuura T., and Ismail A. F. publishes intra-cluster (i.e., no links to other clusters), the remaining four social networks have inter-cluster publications. According to the cluster-based analysis, the social network with the most recent publications ${(DAA}_{It})$ is the purple group consisting of Liu Y. and Zhang Y. (2019.10), while the group with the oldest publications is the gray group consisting of Sourirajan S., Matsuura T., and Ismail A. F. (1995.16). Note that Prof. Sourirajan from the social network passed away in 2022, and Prof. Matsuura has emeritus status. Considering the average global citations per document ${(AGCD}_{It})$ value, the red collaboration network is at the top with a value of 61.56, while the group with the lowest ${AGCD}_{It}$ value is the purple cluster, with a value of 31.16. In terms of the average relative global citations value $\left({ARGC}_{It}\right),$ the first-ranked social network is the red cluster, led by Zhang X., with a value of 1.80. The collaboration network at the bottom of the ${ARGC}_{It}$ value is the purple cluster, with a value of 0.91. When social links are analyzed by scientists, Gao C. has the highest number of links (10), while Wang J. has the highest number of co-authored articles (total link strength) with 92. Yang Y. has the most recent publications (2019.77), while Sourirajan S. has the oldest publications (1976.76). Tang C. Y. is the leading author in ${AGCD}_{It}$ value (105.64), while Liu Y. has the lowest ${AGCD}_{It}$ value with 26.45. In ARGC value, Tang C. Y. is at the top with 2.79, while Liu Y. is at the bottom with 0.83.

### 3.3. Significant Journals

**Figure 6:**
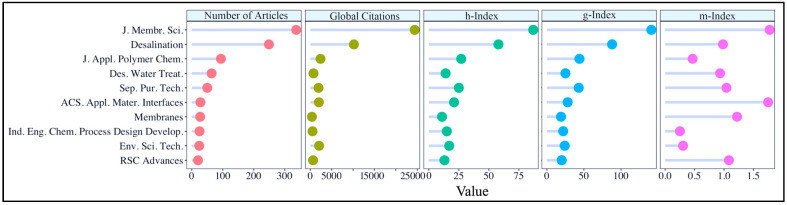
Important metrics of top 10 journals publishing on RO membrane engineering based on the number of articles.

Scientific journals are one of the most important pillars in science to share ideas and scientific findings with the community and the public, to contribute to the accumulation and development of scientific knowledge, and to protect scientific ethics. In addition, researchers can advance their academic careers by publishing in trusted and prestigious journals. Leading scientific sources (top 10) based on the number of publications and the corresponding indicators are given in Figure 6.

The first thing that stands out in Figure 6 is that the *Journal of Membrane Science* and *Desalination* are the main targets of the authors in this collection. In particular, the *Journal of Membrane Science* is the most inclusive journal in the field of RO membrane engineering, reaching the highest values in all criteria (number of published articles = 337, number of global citations = 24,422, *h*-index = 87, *g*-index = 141 and *m*-index = 1.776), which shows that the journal has a wide impact. *Desalination* ranked second in all metrics except *m*-index value (number of published articles = 249, number of global citations = 10,194, *h*-index = 58, *g*-index = 88), while *ACS Applied Materials & Interfaces* was the second journal with the highest *m*-index value (1.75). What if we combine author analyses with journal analyses and examine which scientific journals the top authors tend to publish in? Figure S2 shows the number of articles of the top 10 authors in the top 10 journals in an alluvial graph, and Supplementary Information Note 4 has pertinent remarks.

### 3.4. Essential Affiliations

**Figure 7:**
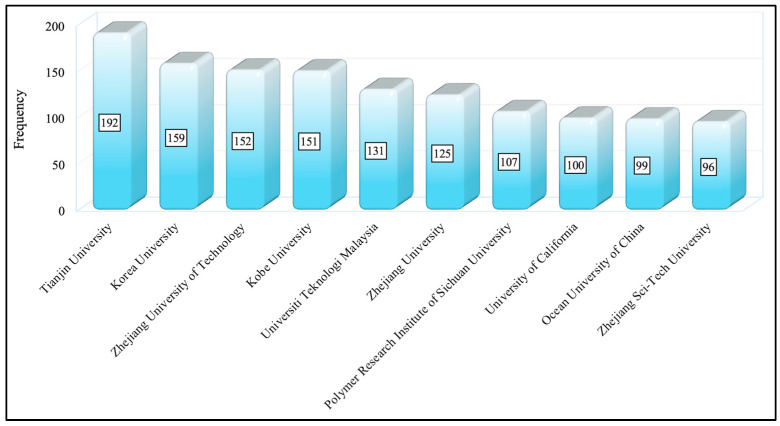
Most relevant affiliations.

Affiliations, such as universities, institutes, or research centers, to which the researchers are associated mostly support their research. It is important to identify significant affiliations in a scientific field to present productive, reliable, reputable, resource-allocating, and supporting affiliations to the readers. The top 10 affiliations in the dataset can be seen in Figure 7. It should be highlighted that the Biblioshiny program counts affiliations by author (i.e., if two authors of a work belong to the same affiliation, Biblioshiny counts this as two affiliations rather than one).

As Figure 7 indicates, Tianjin University (China) is the leading institution publishing on reverse osmosis membrane engineering with a frequency of 192. Korea University (Republic of Korea) ranked second (159), and Zhejiang University of Technology (China) ranked third with a frequency of 152.

### 3.5. Key Articles

**Figure 8:**
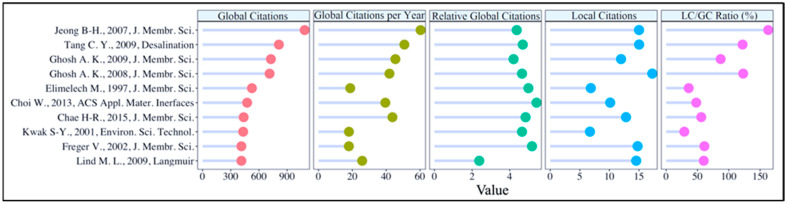
Metrics of top 10 articles (based on global citations) [48,128,200,211,220,294,295,296,297,298] in the reverse osmosis membrane engineering domain.

Presenting important articles in a scientific field to readers is a good way to promote and disseminate influential, groundbreaking, and inspiring work. Figure 8 presents the top 10 authors in the domain based on the number of articles and their corresponding specific metrics.

In Figure 8, the top-ranked paper in three of the five indicators (GC = 1086, LC = 163, ${GCY}_{i}$ = 60.33) is Joeng et al. (2007) with the study titled “Interfacial polymerization of thin film nanocomposites: A new concept for reverse osmosis membranes” [128]. In this research, a novel concept for the formation of mixed matrix reverse osmosis (RO) membranes consisting of nanocomposite thin films by in situ interfacial polymerization on porous polysulfone supports is presented. Nanocomposite films containing NaA zeolite nanoparticles exhibit surface morphologies like commercial RO membranes, while nanocomposite membranes have smoother, more hydrophilic and negatively charged surfaces. At the highest nanoparticle loadings, nanocomposite films are characterized by higher water permeability and equivalent solvent retention. This novel technology offers new possibilities for customizing the performance and material properties of RO membranes. The leading publication on the LC/GC ratio (17.25%) is conducted by Ghosh et al. (2008) titled “Impacts of reaction and curing conditions on polyamide composite reverse osmosis membrane properties” [295]. In this study, the effects of organic solvent properties, reaction conditions, and curing conditions on the performance of polyamide composite reverse osmosis (RO) membranes were investigated. It was found that MPD diffusion affects water permeability, MPD solubility affects crosslinking, and water permeability is most strongly related to film structure, while salt rejection is most strongly related to film thickness and morphology. High-performance RO membranes were obtained by selecting high-surface-tension, low-viscosity solvents and optimizing curing temperature and time. The paper with the highest relative global citations (${RGC}_{i_{y}}$) (5.42) value is “Layer-by-Layer Assembly of Graphene Oxide Nanosheets on Polyamide Membranes for Durable Reverse-Osmosis Applications” by Choi et al. (2013) [296]. In this research, it was demonstrated that the properties of graphene oxide (GO) nanosheets, such as high hydrophilicity, chemical resistance, and fast water permeability, can be used to improve the fouling and chlorine resistance of polyamide (PA) thin-film composite (TFC) membranes. GO coating improved the antifouling performance by increasing surface hydrophilicity and reducing surface roughness. Furthermore, the chemically inert nature of GO nanosheets significantly reduced membrane degradation by acting as a chlorine barrier.

### 3.6. Notable References

**Figure 9:**
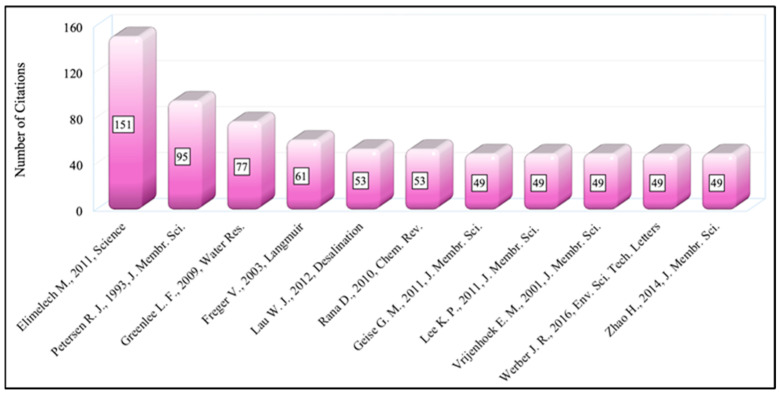
Most cited references (top 10) [138,300,301,302,303,304,305,306,307,308,309] by the RO membrane engineering community.

The use of bibliographies (references) in scientific articles is a sine qua non of scientific writing in terms of respecting the work of the original authors, increasing the reliability and validity of the article, following the source of the information presented, and demonstrating scientific knowledge. The most cited references (top 10) by articles in the field of RO membrane engineering have been revealed in Figure 9. Most cited references refer to the number of citations obtained by a reference (a document that appears in at least one of the bibliographies of the articles) from documents in the dataset [299]. Note that because numbers 7–11 have the same number of citations, the figure includes 11 references.

As Figure 9 indicates, the most commonly referenced, and mostly cited paper by the RO membrane engineering community is written by Elimelech M. and Phillip W. (2011) titled “The Future of Seawater Desalination: Energy, Technology, and the Environment” [300]. This review article has been cited 151 times. In this paper, the authors discuss the potential reductions in energy demand by innovative seawater desalination technologies, the potential role of advanced materials and new technologies in enhancing performance, and the long-term viability of desalination as a technological solution to worldwide water scarcity. The second most referenced paper is also a review paper with 95 citations by Petersen R. J. (1993) titled “Composite reverse osmosis and nanofiltration membranes” [301]. The review article covers the design and performance of composite membranes used in reverse osmosis and nanofiltration procedures. The third-ranked document is also a review article titled “Reverse osmosis desalination: Water sources, technology, and today’s challenges” written by Greenlee et al. (2009) [302]. This review paper provides detailed information about the RO desalination method, with a simple comparison of seawater and brackish water RO systems and their similarities and differences in process development. It also covers essential RO process parameters, as well as changes caused by feed water properties.

### 3.7. Text Mining Results

**Figure 10:**
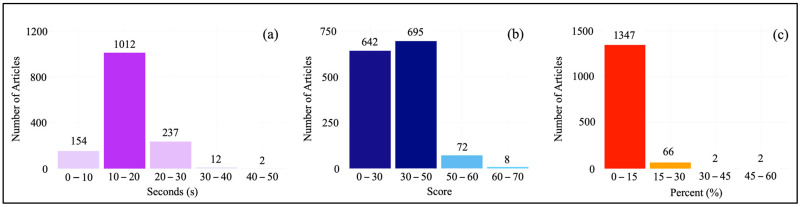
Text mining on abstracts of the articles: (**a**) Reading time score, (**b**) Flesch reading ease score, and (**c**) technical term density ratio (%).

Applying reading time score and Flesch reading ease score analyses to the abstracts of articles can help assess and understand the nature of the dataset. The reading time score provides an important guideline for time management, while the readability score reveals the difficulty of understanding the content. The results are visualized in Figure 10a and Figure 10b, respectively. When the Flesch reading ease scores were analyzed (Figure 10a), it was found that most of the abstracts had scores of 0–30 (difficulty: very difficult, grade level: graduate) or 30–50 (difficulty: difficult, grade level: college) (642 and 695 articles, respectively). Having such high Flesch reading ease scores may indicate that the abstracts contain high technical detail and complexity. This provides in-depth information for academic and professional-level readers. Detailed and technical language can help to fully understand and evaluate the topic. However, there may be some drawbacks to having high Flesch scores, as it can make the articles difficult to understand for a wider audience. Non-academic readers or newcomers to the subject may find it difficult to understand. Hard-to-read texts require more time and effort from readers. This can be a disadvantage for those looking for quick information. More difficult and complex texts may prolong the time it takes readers to absorb information and slow down the research process. We believe that academic articles should strive to achieve a balance between readability and the target audience, so they can reach as wide an audience as possible while providing in-depth information. We acknowledge the limitations of the readability index (FRES) and reading time score metrics used in our study (these metrics are only based on the text-based information). Our method is somewhat straightforward and only applied to the abstracts of the articles. The readability of scientific articles (full body) may not be fully assessed by these metrics alone. For example, technical terms, the reader’s background knowledge and the impact of elements outside the text, such as images, tables, or equations, can also influence the readability of an article. Our findings are limited to the metrics provided by the available methods and applied to specific fields of the articles (abstracts only). However, in the future, new metrics and methods can be developed to better capture the complexity and terminological density of scientific articles. Although the development of these methods is not the subject of this paper, we have performed a technical term density (TTD) assessment on the abstracts in our collection as an example for future work. Figure 10c shows the technical term density distribution of article abstracts. As the figure highlights, in most of the articles (1347), the proportion of technical terms in all terms was measured as low as 0 to 15%. Although FRES grades were found to be very difficult or difficult, TTD results show that the technical term content of the articles is not at a very high level. This indicates that the overall language and structure of the article are quite complex due to complex sentence structures, long sentences, or advanced vocabulary. Only four abstracts were high in terms of technical term content (two articles had 30–45% and two articles had 45–60%). However, it was noticed that the abstracts of these four articles were short. The average percentage of technical terms in abstracts was found to be ~9. We hope that the information and methods provided in this study will lead the way for future studies to determine the readability and reading time score metrics of scientific articles in a specific domain.

**Figure 11:**
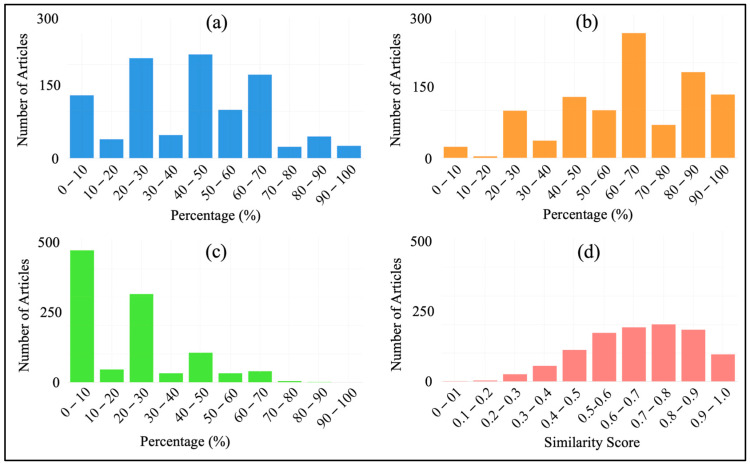
Overlap percentages of author keywords in (**a**) titles, (**b**) abstracts, (**c**) overlap percentage, (**d**) cosine distance scores between author keywords and extracted keywords by Gemini.

Indexers and search engines use keywords as a method to locate appropriate documents. Readers will be able to identify your journal manuscript if database search engines can locate it. This will make more people read your work, which will probably result in more citations. But keywords need to be selected properly to be successful [310]. Some properties of keywords are: to maximize search engine optimization, keywords must be clear and significant, differentiate between “narrow” and “broad” phrases, and be limited in quantity to prevent “keyword spamming” [311]. Keywords serve as an addition to the information supplied in the title. As such, it is not considered standard practice to include words or terms in the title as keywords because relevant indexes will automatically include the words in the title of the article. As a result, keywords and phrases can be thought of as further guidance. To improve searching, authors might find it helpful to provide original keywords. These might be used in addition to, but not in place of, the keywords included in the abstract and title [312]. After all these constraints, an evaluation was carried out to understand what percentage of author keywords the title and abstract sections contain and how RO membrane engineers are successful in finding appropriate keywords (Figure 11a,b). We also used Gemini LLM to extract keywords from abstracts and compared the results with the author keywords of the articles. In this analysis, we aim to present whether the authors had left the keyword selection to an artificial intelligence algorithm and how close (exact match and cosine distance score) they would have gotten to the keywords they selected. Additionally, this investigation allows us to understand how relevant the main ideas or themes of the articles are to the author’s keywords (Figure 11c,d). Since some articles do not contain author keywords, these analyses were conducted on 1033 data instances.

As can be understood from Figure 11a,b, authors of RO membrane engineering articles use the same keywords in the title and abstracts as they use in the author keywords section. This can lead to several problems, such as the lack of highlighting of key topics, the inability to find the desired findings in the search engine results of academic databases and limiting the contribution to the academic field. Figure 11a shows the distribution of the occurrence of the words in the author keywords section in the titles. On average, ~40% of the words in the author keywords section of RO membrane engineering articles are also present in the titles. Figure 11b shows the distribution of the occurrence rate of the words in the author keywords section in the abstracts. This value is up to ~60% in abstracts. All these percentages show that RO membrane engineers should be more careful in choosing necessary keywords.

In Figure 11c, exact match measurement is a very strict metric as it measures keyword-keyword matching; it matters how many words the keyword consists of (word length), and it only accepts words that are exactly the same as a match. Cosine distance is a more flexible similarity measure than exact match. It takes into account the frequency and co-occurrence patterns of words, is not affected by word order, and is independent of how many words the keyword consists of (word length). When exact match values in Figure 11d are analyzed, it is obvious that Gemini was not successful enough in the process of keyword extraction from abstracts. The average percentage of overlapping keywords is 16.50%. In 465 articles, only <10% of keywords could be extracted from abstracts. The highest value achieved by Gemini was 80% for 1 time. In cosine distance values in Figure 11d, an average value of 0.67 was achieved, which indicates Gemini was more successful in capturing some keywords on a word-by-word basis, even if it could not extract the exact content of the keywords. The lowest CD score was found in 1 article with 0.05 (almost all keywords were detected on a word-by-word basis). It was found that Gemini could not extract any keywords contained in author keywords in 69 abstracts (1.0 cosine distance score).

By analyzing the abstracts of a group of articles, readers can understand the overall tone, tell whether the article is positive, negative, or neutral, whether it is written in objective or subjective language, and whether it conveys the author’s emotional expression, bias, or personal opinions. Figure S3 indicates the sentiment, subjectivity, and emotion analyses results of the abstracts in the collection conducted with Gemini.

Figure S3a shows the sentiment results of the abstracts. Most of the abstracts (1054) have a positive perception of the produced membranes or experimental results. Research with positive sentiment is certain to herald successful applications or new developments in the field of RO membrane engineering. It is clear from these results that researchers have a positive attitude toward new technologies or improvements to enhance the performance of RO membranes. In addition, some researchers may respond to the needs of the industry, which may increase the positive sentiment scores. A total of 352 articles have a neutral tone. This score indicates that these articles are not written with a positive or negative reflecting perception. Eleven abstracts have negative sentiments, which means these papers may offer a critical perspective on the design and performance of RO membranes, which may be aimed at questioning the current technology to achieve better results. The authors may have encountered some challenges in the development of RO membranes, such as the properties of membrane materials, water transit mechanisms, and factors that affect the performance of the membrane. Some research may contain negative sentiment as it emphasizes factors that negatively affect the performance of RO membranes. Also, researchers may be working on new technologies and materials to develop future RO membranes. Therefore, critical opinions may have been expressed about existing membranes.

In Figure S3b, the objective writing is dominant (1391 abstracts). This result reflects the authors’ impartial narrative, free from personal opinions. This is already considered a form of academic expression expected of scientists and is a requirement of professionalism. Twenty-six abstracts have subjective language. The authors of these articles should take care to use objective language in their articles, avoid personal opinions, and act in accordance with a scientific style of expression.

As can be read from Figure S3c, all the abstracts (1417) have neutral emotion, which indicates that authors avoided reflecting personal emotions when writing the abstracts. It is important that authors present research results in an emotionally neutral way so that the scientific community can better evaluate the studies and trust the results.

## 4. Conclusions

Reverse osmosis, a technology in which a semi-permeable membrane allows only water molecules to pass through, is now recognized as the most advanced and optimal desalination method. Since Prof. Sourirajan and Prof. Loeb announced the first cellulose acetate membrane at UCLA in 1960, academia and the private sector have focused on producing membranes with high salt rejection capacity, low energy consumption, long lifetime, high flow rate, chemical and fouling resistance, low operating cost, high mechanical strength, and environmental friendliness. This scientific field is known as membrane engineering. 1424 articles downloaded from the Scopus database on 11 March 2024 were examined. After the first publication in 1964, the topic gained popularity especially after 2009. In 2023, 105 articles were published and 8 articles published in 2007 had the highest impact. Membranologists mainly focused on thin-film composite (TFC) polymeric materials (550 papers). The use of nanomaterials and polymers in membrane engineering is widespread (821 papers). Issues such as fouling, biofouling, and scaling are an important focus (324 papers). Wang J. is the author of the most papers in the field with 73 papers, but Gao C. stands out in other metrics (*h*-index = 34, *g*-index = 60, global citation = 3664). *Journal of Membrane Science* is the journal most favored by authors (337 articles). Tianjin University (China) is the leading institution with 192 papers on reverse osmosis membrane engineering. Joeng et al. (2007) ‘Interphase polymerization: A new concept for reverse osmosis membranes’ ranks top in three indicators (global citation = 1086, local citation = 163, annual global citation = 60.33). The most cited paper (151 times) by the RO membrane engineering community was written by Elimelech M. and Phillip W. (2011). Most of the abstracts are written objectively and emotionally neutral. We anticipate that the results of this study will help RO membrane engineering researchers guide their own efforts and establish more successful research methodologies. The information provided in this article can also help new researchers enter the field by distributing research money, providing training programs, and finding new topics of research.

## Figures and Tables

**Table 1:** Keywords used together with the dataset from the Scopus database.

membrane fabrication, membrane preparation, membrane synthesis, interfacial polymerization *,
novel membrane fabrication, state-of-the-art membrane, nanocomposite membrane fabrication,
Thin-film composite membrane fabrication, phenylenediamine and trimesoyl chloride,
membrane engineering, polyamide membrane, cellulose acetate membrane, robust membrane,
hollow fiber membrane, surface coating *, surface modification *, nanomaterial deposition *,
functionalized nanomaterials *, layer-by-layer *, surface grafting *, membrane crosslinking,
surface modified reverse osmosis membrane, polyamide layer regeneration *,
polyamide layer regenerated *, polyamide layer reformation *, polyamide layer reformed *,
tailored *, modification *, coated *, modified *, engineered surface *

* Additionally used the AND operator and “membrane” term to ensure coherent results.

## Data Availability

The raw data supporting the conclusions of this article will be made available by the authors on request.

## Supplementary Materials

The following supporting information can be downloaded at: https://www.mdpi.com/article/10.3390/membranes14120259/s1. Figure S1: Page count, reference count, and citation count distributions of the articles in the collection, Figure S2: Alluvial representation of Top 10 authors publishing in Top 10 journals, Figure S3: (a) Sentiment, (b) subjectivity and (c) emotion analyzes results of the RO membrane engineering articles abstracts. Table S1: Flesch Reading Ease score index interpretation table; Table S2: Essential information of the dataset. All references are cited in the main text.
