# Supplementary material for: Reverse Osmosis Membrane Engineering: Multidirectional Analysis Using Bibliometric, Machine Learning, Data, and Text Mining Approaches
Source: Membranes (Basel). 2024 Dec 6;14(12):259. doi: 10.3390/membranes14120259 (PMC11677704; doi:10.3390/membranes14120259)
Supplement: Supplementary file 1 [file membranes-14-00259-s001.zip › membranes-3330792-supplementary.pdf]

# Supplementary Information

## Reverse osmosis membrane engineering: multidirectional analysis using bibliometric, machine learning, data and text mining approaches

**Ersin Aytaç[1,2], Noman Khalid Khanzada[3], Yazan Ibrahim[3,4], Mohamed Khayet[1,5*], Nidal Hilal[3*]**

[1] Department of Structure of Matter, Thermal Physics and Electronics, Faculty of Physics, University Complutense of Madrid, Avda. Complutense s/n, 28040 Madrid, Spain

[2] Department of Environmental Engineering, Zonguldak Bülent Ecevit University, 67100 Zonguldak, Türkiye

[3] NYUAD Water Research Center, New York University Abu Dhabi, P.O. Box 129188, Abu Dhabi 129188, United Arab Emirates.

[4] Chemical and Biomolecular Engineering Division, New York University, Brooklyn, New York 11201, United States

[5] Madrid Institute for Advanced Studies of Water (IMDEA Water Institute), Avda. Punto Com N◦ 2, 28805 Alcalá de Henares, Madrid, Spain

[*] Corresponding authors:

**Mohamed Khayet** khayetm@fis.ucm.es

**Nidal Hilal** nidal.hilal@nyu.edu

**Supplementary Note 1**

Global citations (GC), (Total Citations (TC) in the Biblioshiny), are the total number of times an item has been cited in a scientific repository (the Scopus database in this study). Local citations (LC) are the number of citations made by other authors for a certain article inside the obtained collection [1].

The compound annual growth rate ($CAGR_C$), (annual growth rate in Biblioshiny), is a measure of a collection's average yearly growth rate over a particular period (Eq. S1) [2]:

$$CAGR_C\ (\%) = \left( \left( \frac{nd_{C_l}}{nd_{C_f}} \right)^{\frac{1}{\left(y_{C_l} - y_{C_f}\right)}} - 1 \right) x\ 100 \tag{S1}$$

where $nd_{C_f}$ reflects the total number of articles from the first year of the collection, $nd_{C_l}$ reflects the total number of articles from the final year of the collection, $y_{C_f}$ reflects the first year of the collection, and $y_{C_l}$ represents the last year of the collection.

The co-authors per document index ($cAD_C$) of the collected dataset is calculated as follows (Eq. S2) [3];

$$cAD_C = \frac{na_C}{nd_C} \tag{S2}$$

where $na_C$ is the overall number of authors in the collection (repeated author names are included) and $nd_C$ is the overall number of documents in the collection.

The mean (average) age of documents in a collection ($DAA_C$) or an item ($DAA_{It}$) is (defined as the average publication year in VOSviewer software) (Eq. S3) [4].

$$DAA_C = \frac{1}{nd_C} \sum_{C_i=1}^{C_n} a_{C_i} \text{ or } DAA_{It} = \frac{1}{nd_{It}} \sum_{It_i=1}^{It_n} a_{It_i} \tag{S3}$$

being, $a_{C_i}$ indicates the age of document $i$, which can be calculated as ($y_C - y_i$), $y_C$ is the year that the dataset was collected and $y_i$ indicates the publication year of the document. $a_{It_i}$ indicates the age of document $i$ of the item, which can be estimated as ($y_C - y_{It_i}$), $y_{It_i}$ is the publication year of this document, and $nd_{It}$ represents the total number of documents of the item.

By using the following calculation, the average GC per document indicator of the dataset ($AGCD_C$) or an item ($AGCD_{It}$) (average citations in VOSviewer and average citations per doc in Biblioshiny) (Eq. S4) [5].

$$AGCD_C = \frac{1}{nd_C} \sum_{i=1}^{nd_C} GC_i \text{ or } AGCD_{It} = \frac{1}{nd_{It}} \sum_{It_i=1}^{It_n} GC_{It_i} \tag{S4}$$

where, $GC_i$ depicts the GC of document $i$ in the collection and $GC_{It_i}$ depicts the GC of document $i$ of the item.

The following equation (Eq. 5) gives the $AGCD_{C_y}$ value of the dataset which is the average GC per document published in the corresponding year (mean times cited per article in Biblioshiny) (Eq. S5) [5]:

$$AGCD_{C_y} = \frac{1}{nd_{C_y}} \sum_{i_y=1}^{nd_{C_y}} GC_{i_y} \quad \text{(S5)}$$

being, $GC_{i_y}$ is the GC of document $i$ published in year $y$ and $nd_{C_y}$ is the number of documents in the dataset published in year $y$.

The $AGCD_{C_y}$ value can be normalized to give the average normalized global citations per documents published in the corresponding year ($ANGCD_{C_y}$) value of the collection (TC per year in Biblioshiny) and can be calculated as in Eq. S6 [6]:

$$ANGCD_{C_y} = \frac{AGCD_{C_y}}{a_{C_y}} \quad \text{(S6)}$$

In here, $a_{C_y}$ is the age of any document used in $AGCD_{C_y}$ calculation.

The $GCY_i$ value which is global citations per year of document $i$ (TC per year in Biblioshiny) can be determined as in Eq. S7 [3]:

$$GCY_i = \frac{GC_i}{a_{C_i}} \quad \text{(S7)}$$

In here, $GC_i$ is the global citations value of $i$.

The relative global citations of any document ($i$) published in a particular year ($RC_{i_y}$) (normalized TC in Biblioshiny) can be calculated with the following equation (Eq. S8) [7]:

$$RGC_{i_y} = \frac{GC_{i_y}}{\sum_{i_y=1}^{nd_{C_y}} GC_{i_y} \Big/ nd_{C_y}} \quad \text{(S8)}$$

being, $GC_{i_y}$ is the number of global citations of $i$.

The average relative global citations ($ARGC_{It}$) value is used in order to correct the problem of differences in citation counts due to the age of a document and brings a deeper understanding of the impact of an item (average normalized citations in VOSviewer software) is calculated as in Eq. S9 [8]:

$$ARGC_{It} = \frac{1}{nd_{It}} \sum_{i=1}^{nd_{It}} RGC_{It_{i_y}} \quad \text{(S9)}$$

being, $RGC_{It_{i_y}}$ is the relative global citations of $i$ of an item published in a particular year $y$.

The international co-authorship ($IcA_C$) ratio of the dataset, can be calculated by the following equation (Eq. S10) [4]:

$$IcA_C\ (\%) = \frac{nmc_C}{nd_C}\ x\ 100 \quad \text{(S10)}$$

where, $nmc_C$ are the publications with affiliations from at least two different countries.

Eq. 11 produces the fractionalized value of an author ($AF_{au}$) which is a metric that evaluates an author's credit by distributing the contribution of each article evenly across the authors and summing it (Eq. S11) [9]:

$$AF_{au} = \sum_{i=1}^{m} \frac{1}{na_{a_i}} \quad \text{(S11)}$$

In here, $na_{a_i}$ is the number of co-authors in $i$ of an author and $m$ is the number of articles of an author.

The $h$-index is an indicator of quantity (number of publications) and impact (number of citations) of an item in a single statistic and is described as an item with $m$ papers have at least $h$ citations each of them and the other $(m - h)$ papers have fewer than $h$ citations each [10]. When an item's citations are sorted in decreasing order ($c_i$), the $h$-index may be calculated using Eq. S12 [11].

$$\max_h \sum_{i=1}^{h} c_i\ \geq h \quad \text{(S12)}$$

The $m$-quotient (denoted $m$-index in Biblioshiny) accounts for an item's academic career longevity by dividing the $h$-index by the number of years from first publication. The calculation of $m$-index is as in Eq. S13 [12].

$$m - index = \frac{h{-}index}{y_{It_l} - y_{It_f}} \quad \text{(S13)}$$

being, $y_{It_l}$ and $y_{It_f}$ are the last and first year of publication of the item respectively. However, in Biblioshiny tool the last publication year of the authors ($y_{It_l}$) is set to the year the dataset downloaded ($y_C$).

The $g$-index refers to the largest number $g$ of scientific works that have achieved at least $g^2$ citations [13]. When the citations of an item are ranked in the decreasing order ($c_i$), the $g$-index of can be found as follows (Eq. S14) [14];

$$\max_g \sum_{i=1}^{g} c_i\ >\ g^2 \Leftrightarrow \max_g \frac{1}{g} \sum_{i=1}^{g} c_i\ >\ g \quad \text{(S14)}$$

For the Flesch Reading Ease Score (FRES) (readability index) and reading time score Textstat package in Python was employed. Textstat is a user-friendly tool for computing statistics that helps assess text-based information [15]. Rudolph Flesch developed the FRES metric in the 1940s, and it is based on average sentence length and average word length. The resulting score runs from 0 to 100, with lower scores suggesting higher reading difficulty [16]. FRES value of a paragraph can be calculated as following Eq. S15 [17].

$$FRE\ Score = 206.835 - \left(1.015\ x\ \frac{words}{sentence}\right) - \left(84.6\ x\ \frac{syllabels}{word}\right) \tag{S15}$$

Table S1 presents an explanation of the generated scores [18, 19].

**Table S1.** Flesch Reading Ease score index interpretation table

| FRES | Difficulty | Grade Level |
|---|---|---|
| 90 – 100 | Very Easy | 5th |
| 80 – 90 | Easy | 6th |
| 70 – 80 | Fairly Easy | 7th |
| 60 – 70 | Average (Plain English) | 8th - 9th |
| 50 – 60 | Fairly Difficult | 10th - 12th |
| 30 – 50 | Difficult | Collage |
| 0 – 30 | Very Difficult | Graduate |

The Textstat package assumes a reading time score of 14.69 ms per character (spaces are not counted as characters) based on the article entitled "Data from eye-tracking corpora as evidence for theories of syntactic processing complexity" [20].

The technical term density percentage (TTD %) calculation was performed in Python. Briefly, the importance of terms was calculated using Term Frequency-Inverse Document Frequency (TF-IDF), more about TF-IDF can be found elsewhere [21]. The terms with the highest TF-IDF score were identified as technical terms ($TT$) (top 50) with some manual refinements. The TTD percent of an abstract can be calculated with Eq. S16;

$$TTD\ (\%) = \frac{Number\ of\ TT\ in\ the\ abstract}{Total\ words\ in\ the\ abstract}\ x\ 100 \tag{S16}$$

For the keyword extraction, sentiment, emotion, and subjectivity analysis, Google Gemini LLM (gemini-1.5-flash) was used. Gemini is a very powerful LLM built by Google that is trained across video, text data, audio, and picture, to perform both high generalist capabilities across modalities and cutting-edge comprehension and reasoning performance in each domain [22]. To use Google Gemini, an API was key provided from Google AI Studio. Then the following steps were coded:

defining the API key, generation config and safety settings, configuring the Google Gemini API, selecting the model (gemini-1.5-flash-002), function of the analysis used, saving and loading progress functions, storing analysis results in data frame and saving results. Keyword extraction is the technique of automatically extracting relevant words and phrases from documents using predetermined rules or models created in the field of text mining. Keywords, which are the fundamental components expressing the essence or theme of a document, play an important role in tasks such as text summarization, text categorization, and clustering. In corpus linguistics, keyword extraction is a popular method for investigating language trends [23, 24]. In keyword extraction, the similarity of the extracted keywords with the author keywords is evaluated in two ways, exact match, and cosine distance (CD) score. The exact match process is case insensitive. The pre-processing step in cosine similarity includes converting text to lower case and removing non-alphanumeric characters.

Mathematically, exact match (EM) is expressed as follows (Eq. S17);

$$EM\ (\%) = \left(\frac{|A \cap B|}{|B|}\right) x\ 100 \qquad \text{(S17)}$$

where $A$ is the set of automatically extracted keywords, $B$ is the set of author keywords, $|A \cap B|$ is the number of elements in the intersection of two sets. This metric takes a value between 0 % and 100 % where 0 % means no overlap at all and 100 % is full overlap.

Cosine similarity metric that measures the angle between two vectors. In this context, it represents sets of keywords in vector space and measures the similarity between them. The cosine similarity metric between two vectoral forms of 2 data $(u, v)$, being $u = (u_1, u_2, \dots\dots, u_m) \in \mathbb{R}^m$ and $v = (v_1, v_2, \dots\dots, v_m) \in \mathbb{R}^m$, is (Eq. S18) [25, 26];

$$CS = \frac{u.v}{|u||v|} = \frac{\sum_{i=1}^{m} u_i\, v_i}{\sum_{i=1}^{m} u\, \sum_{i=1}^{n} v_i^2} \qquad \text{(S18)}$$

Cosine similarity takes a value between -1 and 1. A value of 1 means exact similarity (vectors point in the same direction), a value of -1 means complete difference (vectors point in opposite directions), and 0 indicates no similarity.

Cosine distance ($CD$) of two data points can be calculated as $CD = 1 - CS$ [27], which results in a value between 0 and 2, indicating similarity and dissimilarity respectively.

Sentiment analysis, also known as opinion mining or opinion analysis, is an important study field in NLP. It is aimed to automatically extract and evaluate sentiments and viewpoints of users from text mainly as positive, negative, or neutral [28, 29]. Emotion analysis is to seek out users'

emotional states such as anger, love, joy, etc. [30]. Although the terms emotion and sentiment are synonyms and can be used interchangeably, they have different meanings in NLP. The term emotion refers to more distinct, powerful, and personal feelings, such as love, fear, etc. whereas sentiment is confined to general perception [31, 32]. Subjectivity analysis is employed to classify textual data as if it is carrying an opinion (subjective) or it is factual (objective) [33].

**Supplementary Note 2**

Table S2 indicates the essential information about the RO membrane engineering collection.

**Table S2.** Essential information of the dataset

| **Information** | **Outcome** |
|---|---|
| Timespan | 1964:2023 |
| Number of Journals | 225 |
| Compound Annual Growth Rate ($CAGR_C$) (%) | 8.21 |
| Document Average Age ($DAA_C$) | 14 |
| Average Citations per Document ($AGCD_C$) | 41.75 |
| Total References | 38424 |
| Author's Keywords | 1987 |
| Number of Authors | 3110 |
| Authors of Single-Authored Documents | 39 |
| Single-Authored Documents | 42 |
| Co-Authors per Document ($cAD_C$) | 4.79 |
| International Co-Authorships Ratio ($IcA_C$)( (%) | 21.07 |

**Supplementary Note 3**

The distribution of the number of pages, number of references and number of citations of the articles in the data set is shown in Fig. S1

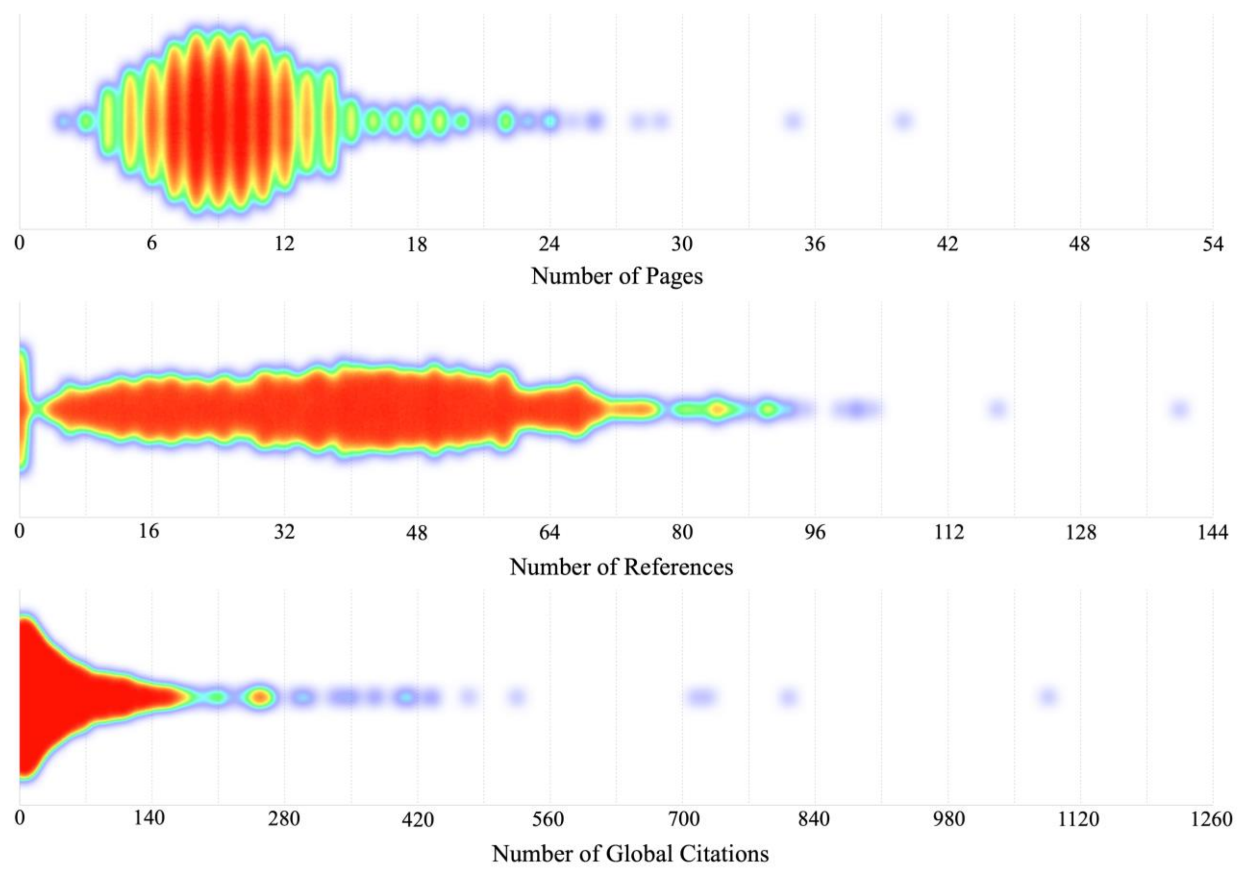


**Figure S1.** Page count, reference count, and citation count distributions of the articles in the collection.

Fig. S1 shows that most of the articles in the collection (note that 397 articles do not have page counts) have page counts between 7-12 (average ~10). The most common article length (mode) is 8 pages (150 articles). The shortest articles have 2 pages, while the longest article has 40 pages entitled "Selective properties of cellulose acetate membranes towards ions in aqueous solutions" written by T. D. Hodgson (1970) [34]. In this study briefly, the selective removal of various salts from aqueous solutions using reverse osmosis was explored. A basic theoretical model is used to demonstrate for the salts tested, there is a distinct link between the rejection of one salt and that of another for any membrane "tightness". RO membrane engineering studies use ~38 references on

average. 55 articles do not include any references. It should be noted that some older articles were written without references, or the reference sections were not recorded in the Scopus database. The article with the highest number of references (140) is the article by Shen et al. (2023) named "When self-assembly meets interfacial polymerization" [35]. In this study, an ultra-permeable polyamide reverse osmosis membrane with crumpled surface morphology and increased free volume was created using interfacial polymerization reaction with the addition of a self-assembled surfactant micellar system. Multiscale simulations were used to clarify the principles governing crumpled nanostructure development. When the global citation numbers of the articles are analyzed, it is seen that 75 articles are missing citations. Either these articles have not yet been cited by any source or citation information has not been recorded in the database. It was noticed in the remaining 1349 articles, that the average global citation value was ~42 and most of the articles (53) received 1 citation (mode value). The most cited article is "Interfacial polymerization of thin film nanocomposites: A new concept for reverse osmosis membranes" with 1086 global citations by Jeong et al. (2007) [36]. The researchers present a novel idea for generating mixed matrix RO membranes via interfacial polymerization of nanocomposite thin films in situ on porous polysulfone substrates. These nanocomposite films contain NaA zeolite nanoparticles distributed among polyamide films that are 50-200 nm thick. This paper then will be referred to as the first article involving the production of thin film nanocomposite (TFN) membranes.

**Supplementary Note 4**

Fig. S2 shows the number of articles of the top 10 authors in the top 10 journals in an alluvial graph.

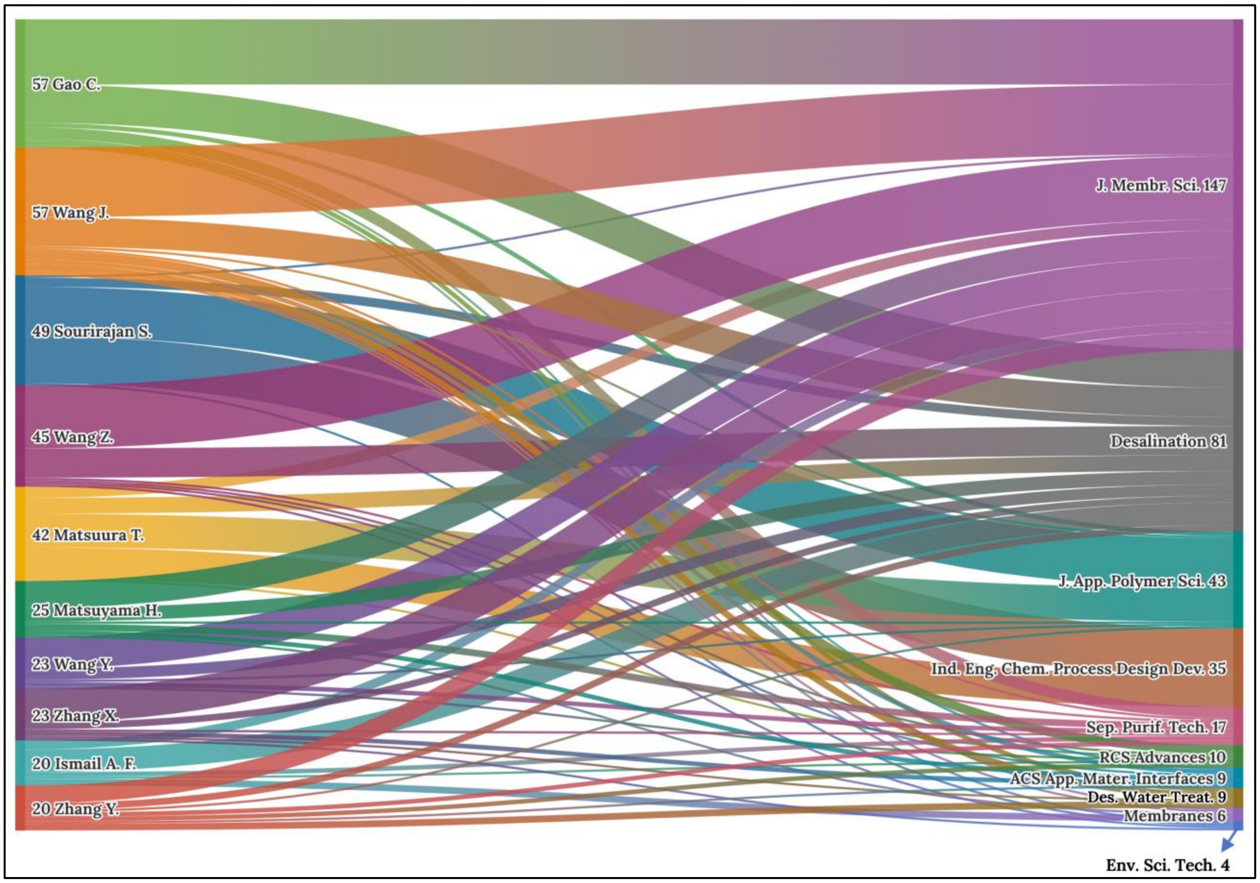


**Figure S2.** Alluvial representation of Top 10 authors publishing in Top 10 journals.

Fig. S2 clearly shows that the top 10 authors have published at least 1 article in the Journal of Membrane Science and Desalination. Wang J. is the most published author in the Journal of Membrane Science with 31 papers. He is followed by Gao C. with 29 papers. Gao C. is also the most published author in Desalination with 17 papers. The journals that Prof. Sourirajan mostly preferred to publish his scientific results were the Journal of Applied Polymer Science (22) and Industrial & Engineering Chemistry Research (21). Prof. Matsuura has also dominantly chosen these two journals to publish his work.

**Supplementary Note 5**

Fig. S3 indicates the sentiment, subjectivity and emotion analyzes results of the abstracts in the collection conducted with Gemini.

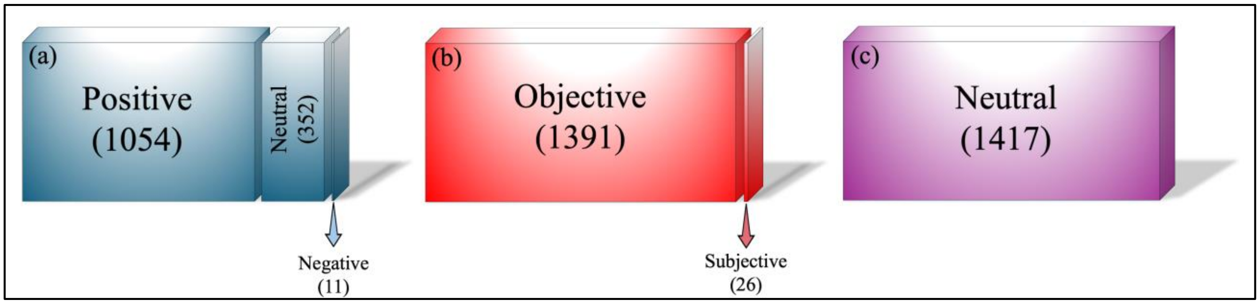


**Figure S3. (a)** Sentiment, **(b)** subjectivity and **(c)** emotion analyzes results of the RO membrane engineering articles abstracts.

**References**


[1] E. Aytaç, M. Khayet, A deep dive into membrane distillation literature with data analysis, bibliometric methods, and machine learning, Desalination 553 (2023) 116482. https://doi.org/https://doi.org/10.1016/j.desal.2023.116482.

[2] B. Niu, H.A. Loáiciga, Z. Wang, F.B. Zhan, S. Hong, Twenty years of global groundwater research: A Science Citation Index Expanded-based bibliometric survey (1993–2012), J. Hydrol. 519 (2014) 966-975. https://doi.org/https://doi.org/10.1016/j.jhydrol.2014.07.064.

[3] E. Aytaç, J. Contreras-Martínez, M. Khayet, Mathematical and computational modeling of membrane distillation technology: A data-driven review, Int. J. Thermofluids 21 (2024) 100567. https://doi.org/https://doi.org/10.1016/j.ijft.2024.100567.

[4] M. Khayet, E. Aytaç, A Glimpse into Dr. Nidal Hilal's Scientific Achievements, J. Membr. Sci. Res. 10(2) (2024) -. https://doi.org/https://doi.org/10.22079/jmsr.2023.1999042.1598.

[5] E. Aytaç, A. Fombona-Pascual, J.J. Lado, E.G. Quismondo, J. Palma, M. Khayet, Faradaic deionization technology: Insights from bibliometric, data mining and machine learning approaches, Desalination (2023) 116715. https://doi.org/https://doi.org/10.1016/j.desal.2023.116715.

[6] S. Ngassa, K. Kilulya, R. Masalu, Scientific Landscape on Phthalates Biodegradation Research: A Bibliometric and Scientometric Study, Tanzania J. Sci. 49 (2023) 842-858. https://doi.org/10.4314/tjs.v49i4.6.

[7] M. Ruiz-Pérez, J.M. Seguí-Pons, X. Salleras-Mestre, Bibliometric analysis of equity in transportation, Heliyon 9(8) (2023) e19089. https://doi.org/https://doi.org/10.1016/j.heliyon.2023.e19089.

[8] L. Liao, L. Quan, C. Yang, L. Li, Knowledge synthesis of intelligent decision techniques applications in the AECO industry, Automat. Constr. 140 (2022) 104304. https://doi.org/https://doi.org/10.1016/j.autcon.2022.104304.

[9] D. Xu, H. Sun, J. Wang, N. Wang, Y. Zuo, A.A. Mosa, X. Yin, Global trends and current advances regarding greenhouse gases in constructed wetlands: A bibliometric-based quantitative review over the last 40 years, Ecol. Eng. 193 (2023) 107018. https://doi.org/https://doi.org/10.1016/j.ecoleng.2023.107018.
[10] C. García-Villar, J.M. García-Santos, Bibliometric indicators to evaluate scientific activity, Radiología (English Edition) 63(3) (2021) 228-235. https://doi.org/https://doi.org/10.1016/j.rxeng.2021.01.002.
[11] R.S.J. Tol, A rational, successive g-index applied to economics departments in Ireland, J. Informetr. 2(2) (2008) 149-155. https://doi.org/https://doi.org/10.1016/j.joi.2008.01.001.
[12] A.A.B. Jamjoom, A.N. Wiggins, J.J.M. Loan, J. Emelifeoneu, I.P. Fouyas, P.M. Brennan, Academic Productivity of Neurosurgeons Working in the United Kingdom: Insights from the H-Index and Its Variants, World Neurosurg. 86 (2016) 287-293. https://doi.org/https://doi.org/10.1016/j.wneu.2015.09.041.
[13] B. Dragović, N. Zrnić, A. Dragović, E. Tzannatos, M.A. Dulebenets, A comprehensive bibliometric analysis and assessment of high-impact research on the berth allocation problem, Ocean Eng. 300 (2024) 117163. https://doi.org/https://doi.org/10.1016/j.oceaneng.2024.117163.
[14] H.H. Lathabai, ψ-index: A new overall productivity index for actors of science and technology, J. Informetr. 14(4) (2020) 101096. https://doi.org/https://doi.org/10.1016/j.joi.2020.101096.
[15] A. Ward, Textstat. https://github.com/textstat/textstat, 2024 (accessed April 22 2024).
[16] C. Ferguson, M. Merga, S. Winn, Communications in the time of a pandemic: the readability of documents for public consumption, Aust. Nz J. Publ. Heal. 45(2) (2021) 116-121. https://doi.org/https://doi.org/10.1111/1753-6405.13066.
[17] D. Zhang, B.E. Earp, E.E. Kilgallen, P. Blazar, Readability of Online Hand Surgery Patient Educational Materials: Evaluating the Trend Since 2008, The Journal of Hand Surgery 47(2) (2022) 186.e1-186.e8. https://doi.org/https://doi.org/10.1016/j.jhsa.2021.03.025.
[18] Z.W. Taylor, Writing Dollars into Sense: Simplifying Financial Aid for L2 Students, Journal of Student Affairs Research and Practice 56(4) (2019) 438-453. https://doi.org/10.1080/19496591.2019.1614937.
[19] B. Ho, E.M. Hong, B.E. Benson, Assessing and Improving the Effectiveness of Online Patient Education Materials on Essential Vocal Tremor: A Comprehensive Evaluation, J. Voice (2024). https://doi.org/https://doi.org/10.1016/j.jvoice.2024.02.021.
[20] V. Demberg, F. Keller, Data from eye-tracking corpora as evidence for theories of syntactic processing complexity, Cognition 109(2) (2008) 193-210. https://doi.org/https://doi.org/10.1016/j.cognition.2008.07.008.
[21] TF–IDF, in: C. Sammut, G.I. Webb (Eds.), Encyclopedia of Machine Learning, Springer US, Boston, MA, 2010, pp. 986-987. https://doi.org/10.1007/978-0-387-30164-8_832.
[22] Gemini Team, Rohan Anil, Sebastian Borgeaud, Y. Wu, J.-B. Alayrac, J. Yu, R. Soricut, J. Schalkwyk, A.M. Dai, A. Hauth, K. Millican, D. Silver, S. Petrov, M. Johnson, I. Antonoglou, J. Schrittwieser, A. Glaese, J. Chen, E. Pitler, T. Lillicrap, A. Lazaridou, O. Firat, J. Molloy, M. Isard, P.R. Barham, T. Hennigan, B. Lee, F. Viola, M. Reynolds, Y. Xu, R. Doherty, E. Collins, C. Meyer, E. Rutherford, Gemini: A Family of Highly Capable Multimodal Models, arXiv (2023).
[23] L.-C. Chen, An extended TF-IDF method for improving keyword extraction in traditional corpus-based research: An example of a climate change corpus, Data Knowl. Eng. 153 (2024) 102322. https://doi.org/https://doi.org/10.1016/j.datak.2024.102322.

[24] L.-C. Chen, K.-H. Chang, An entropy-based corpus method for improving keyword extraction: An example of sustainability corpus, Eng. Appl. Artif. Intel. 133 (2024) 108049. https://doi.org/https://doi.org/10.1016/j.engappai.2024.108049.
[25] S. Liaquat, M.F. Zia, O. Saleem, Z. Asif, M. Benbouzid, Performance analysis of distance metrics on the exploitation properties and convergence behaviour of the conventional firefly algorithm, Appl. Soft Comput. 126 (2022) 109255. https://doi.org/https://doi.org/10.1016/j.asoc.2022.109255.
[26] J.S. Yap, M.H. Lim, L.M. Salman, Improved versatility and robustness of bearing fault detection and diagnostic method for nuclear power plant, Nucl. Eng. Des. 428 (2024) 113474. https://doi.org/https://doi.org/10.1016/j.nucengdes.2024.113474.
[27] Z. He, Y. Lin, Z. Lin, C. Wang, Multi-label feature selection via similarity constraints with non-negative matrix factorization, Knowl-based Syst. 297 (2024) 111948. https://doi.org/https://doi.org/10.1016/j.knosys.2024.111948.
[28] Y. Mao, Q. Liu, Y. Zhang, Sentiment analysis methods, applications, and challenges: A systematic literature review, J. King Saud Univ. Comput. Inf. Sci. 36(4) (2024) 102048. https://doi.org/https://doi.org/10.1016/j.jksuci.2024.102048.
[29] B. Seong, K. Song, Sentiment analysis of online responses in the performing arts with large language models, Heliyon 9(12) (2023) e22457. https://doi.org/https://doi.org/10.1016/j.heliyon.2023.e22457.
[30] F. Naznin, I. Hazarika, D. Laskar, A.K. Mahanta, Mining association between different emotion classes present in users posts of social media, Social Network Analysis and Mining 14(1) (2024) 76. https://doi.org/10.1007/s13278-024-01241-w.
[31] H. Aka Uymaz, S. Kumova Metin, Vector based sentiment and emotion analysis from text: A survey, Eng. Appl. Artif. Intel. 113 (2022) 104922. https://doi.org/https://doi.org/10.1016/j.engappai.2022.104922.
[32] P. Nandwani, R. Verma, A review on sentiment analysis and emotion detection from text, Social Network Analysis and Mining 11(1) (2021) 81. https://doi.org/10.1007/s13278-021-00776-6.
[33] A. Al Hamoud, A. Hoenig, K. Roy, Sentence subjectivity analysis of a political and ideological debate dataset using LSTM and BiLSTM with attention and GRU models, J. King Saud Univ. Comput. Inf. Sci. 34(10, Part A) (2022) 7974-7987. https://doi.org/https://doi.org/10.1016/j.jksuci.2022.07.014.
[34] T.D. Hodgson, Selective properties of cellulose acetate membranes towards ions in aqueous solutions, Desalination 8(1) (1970) 99-138. https://doi.org/https://doi.org/10.1016/S0011-9164(00)82017-2.
[35] Q. Shen, Q. Song, Z. Mai, K.R. Lee, T. Yoshioka, K. Guan, R.R. Gonzales, H. Matsuyama, When self-assembly meets interfacial polymerization, Sci Adv 9(18) (2023) eadf6122. https://doi.org/10.1126/sciadv.adf6122.
[36] B.-H. Jeong, E.M.V. Hoek, Y. Yan, A. Subramani, X. Huang, G. Hurwitz, A.K. Ghosh, A. Jawor, Interfacial polymerization of thin film nanocomposites: A new concept for reverse osmosis membranes, J. Membr. Sci. 294(1) (2007) 1-7. https://doi.org/https://doi.org/10.1016/j.memsci.2007.02.025.
